# Structure-based classification predicts drug response in *EGFR*-mutant NSCLC

**DOI:** 10.1038/s41586-021-03898-1

**Published:** 2021-09-15

**Authors:** Jacqulyne P. Robichaux, Xiuning Le, R. S. K. Vijayan, J. Kevin Hicks, Simon Heeke, Yasir Y. Elamin, Heather Y. Lin, Hibiki Udagawa, Ferdinandos Skoulidis, Hai Tran, Susan Varghese, Junqin He, Fahao Zhang, Monique B. Nilsson, Lemei Hu, Alissa Poteete, Waree Rinsurongkawong, Xiaoshan Zhang, Chenghui Ren, Xiaoke Liu, Lingzhi Hong, Jianjun Zhang, Lixia Diao, Russell Madison, Alexa B. Schrock, Jennifer Saam, Victoria Raymond, Bingliang Fang, Jing Wang, Min Jin Ha, Jason B. Cross, Jhanelle E. Gray, John V. Heymach

**Affiliations:** 1 Department of Thoracic/Head and Neck Medical Oncology, MD Anderson Cancer Center, Houston, TX USA; 2 Institute for Applied Cancer Science, MD Anderson Cancer Center, Houston, TX USA; 3 Department of Individualized Cancer Management, Moffitt Cancer Center, Tampa, FL USA; 4 Department of Biostatistics, MD Anderson Cancer Center, Houston, TX USA; 5 Quantitative Research Computing, MD Anderson Cancer Center, Houston, TX USA; 6 Department of Thoracic and Cardiovascular Surgery, MD Anderson Cancer Center, Houston, TX USA; 7 Department of Thoracic Oncology, West China Medical School, West China Hospital, Sichuan University, Sichuan, China; 8 Department of Bioinformatics and Computational Biology, MD Anderson Cancer Center, Houston, TX USA; 9 Foundation Medicine, Cambridge, MA USA; 10 Guardant Health, Redwood City, CA USA; 11 Department of Thoracic Oncology, Moffitt Cancer Center, Tampa, FL USA

**Keywords:** Targeted therapies, Non-small-cell lung cancer

## Abstract

Epidermal growth factor receptor (*EGFR*) mutations typically occur in exons 18–21 and are established driver mutations in non-small cell lung cancer (NSCLC)^1–3^. Targeted therapies are approved for patients with ‘classical’ mutations and a small number of other mutations^4–6^. However, effective therapies have not been identified for additional *EGFR* mutations. Furthermore, the frequency and effects of atypical *EGFR* mutations on drug sensitivity are unknown^1,3,7–10^. Here we characterize the mutational landscape in 16,715 patients with *EGFR*-mutant NSCLC, and establish the structure–function relationship of *EGFR* mutations on drug sensitivity. We found that *EGFR* mutations can be separated into four distinct subgroups on the basis of sensitivity and structural changes that retrospectively predict patient outcomes following treatment with EGFR inhibitors better than traditional exon-based groups. Together, these data delineate a structure-based approach for defining functional groups of *EGFR* mutations that can effectively guide treatment and clinical trial choices for patients with *EGFR*-mutant NSCLC and suggest that a structure–function-based approach may improve the prediction of drug sensitivity to targeted therapies in oncogenes with diverse mutations.

## Abstract (web summary)

Structural classification of mutations in the epidermal growth factor receptor causing non-small cell lung cancer is a better predictor of patient outcomes following drug treatment than traditional exon-based classification.

## Main

Patients with classical EGFR mutations (L858R or exon 19 deletions (Ex19del)) show marked improvements in clinical outcomes when treated with first-, second- or third-generation tyrosine kinase inhibitors (TKIs)^4–6,11^. Other *EGFR* mutations in the kinase domain (exons 18−21) have also been established as oncogenic drivers of NSCLC^8^. Patients with atypical *EGFR* mutations show heterogeneous and reduced responses to EGFR inhibitors^1,3,7–10^, including osimertinib^12^. Atypical *EGFR* mutations with US Food and Drug Administration (FDA)-approved treatments are *EGFR* S768I, L861Q and G719X, for which afatinib was deemed effective on the basis of retrospective studies^13–15^, and the EGFR/MET bispecific antibody amivantamab for exon 20 insertions (Ex20ins)^16^. There are no clear established guidelines for EGFR TKI treatment for patients with atypical *EGFR* mutations without an FDA-approved TKI, often resulting in patients receiving chemotherapy. Clinical trial design and treatment of patients with atypical *EGFR* mutations often rely on mutated-exon location to predict treatment, although heterogeneity in drug sensitivity across a single exon has been observed^1,8,17^. Therefore, there is an unmet clinical need to establish a system for classifying *EGFR* mutations that is predictive of drug sensitivity and for more robust clinical trial design.

## Clinical outcomes for atypical mutations

**Fig. 1:**
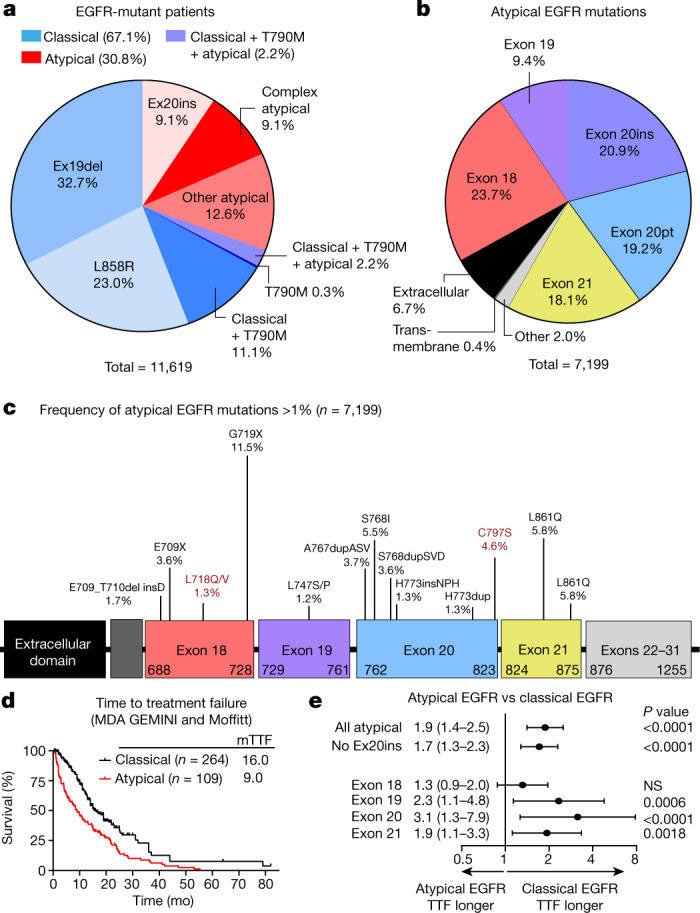
Atypical *EGFR* mutations are associated with worse patient outcomes. **a**, Percentage of patients with NSCLC containing classical and atypical *EGFR* mutations (*n* = 11,619 patients). Classical *EGFR* mutations are L858R, T790M and various Ex19dels (Methods). **b**, Percentage of atypical *EGFR* mutations observed in patients with NSCLC (*n* = 7,199 mutations). Atypical *EGFR* mutations are defined as non-classical, non-synonymous mutations. **c**, Lollipop plot of frequency of atypical *EGFR* mutations in patients with NSCLC (*n* = 7,199 mutations). *EGFR* mutations associated with acquired drug resistance are highlighted in red. **d**, Kaplan–Meier plot of time to treatment failure (TTF) (time from TKI commencement until radiologic progression, discontinuation, or death) of patients with NSCLC tumours containing classical (*n* = 245 patients) or atypical (*n* = 109 patients) *EGFR* mutations after EGFR TKI treatment. **e**, Forest plot of HR calculated from Kaplan–Meier plots of patients with various subsets of atypical mutations or classical EGFR mutations. In **d**, **e**, HR and *P* value were calculated using two-sided Mantel–Cox log-rank tests. Data are HR ± 95% confidence interval. All atypical, *n* = 109; all atypical without Ex20ins, *n* = 97; exon 18, *n* = 29; exon 19, *n* = 22; exon 20, *n* = 41; exon 21, *n* = 18. NS, not significant. Source data.

**Extended Data Fig 1:**
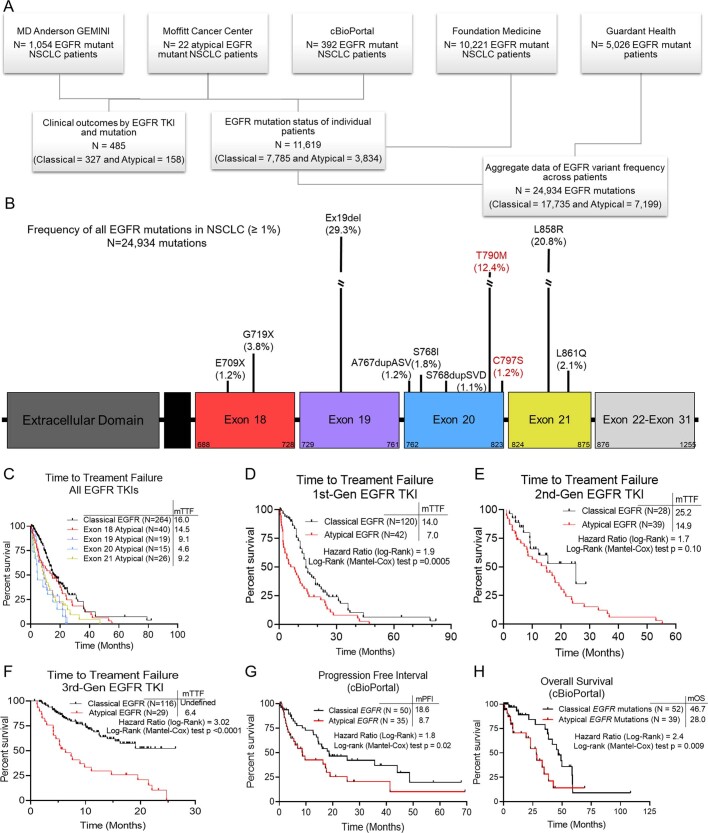
Patients with atypical EGFR mutations have worse clinical outcomes than those with classical EGFR mutations. **a**, Diagram of patient sample sources and types across databases. **b**, Lollipop plot of frequency of all EGFR mutations observed in patients with NSCLC (N=24,934 mutations). *EGFR* mutations associated with acquired drug resistance, as described by the literature, are highlighted in red. **c**–**f**, Kaplan-Meier plot of TTF of patients with NSCLC tumors harboring classical (N=264 patients) or atypical *EGFR* mutations stratified by (**c**) exon after treatment with an EGFR TKI (Exon 18 N= 40, Exon 19 N=19, Exon 20 N=15, Exon 21 N=26), or EGFR TKI class including 1^st^- (**d**), 2^nd^- (**e**), or 3^rd^- (**f**) gen TKIs in MD Anderson GEMINI and Moffitt Cancer center databases. Patients that received prior chemotherapy or immunotherapy were included, but TTF was calculated for first EGFR TKI received. **g**, **h**, Kaplan-Meier plot of PFI (**g**) and OS (**h**) of patients with NSCLC tumors harboring classical (N=50 for PFI and N=52 for OS) or atypical (N=35 for PFI and N=39 for OS) *EGFR* mutations from cBioPortal. Atypical EGFR mutations were limited to mutations in the tyrosine kinase domain, and treatment and stage were unknown. **c**–**h**, HRs and p-values were calculated using two-sided Mantel-Cox, Log-Rank tests Source data.

To characterize the molecular landscape of *EGFR*-mutant NSCLC, we used 5 patient databases with genomic profiling (Methods), representing 16,715 patients with *EGFR*-mutant NSCLC. There were 11,619 patients in whom primary and/or co-occurring mutations were recorded on a per-patient basis (Extended Data Fig. 1a). Among those patients, 67.1% had classical *EGFR* mutations (L858R and/or Ex19del with or without T790M); 30.8% had atypical *EGFR* mutations, including Ex20ins (9.1%), atypical mutations (12.6%), or a complex mutation including an atypical mutation (9.1%); and 2.2% had a classical mutation with T790M and an atypical mutation (Fig. 1a, Extended Data Fig. 1b). Atypical *EGFR* mutations (*n* = 7,199) occurred primarily in exons 18 (23.7%) and 20 (20.9% insertions and 19.2% point mutations; Fig. 1b). Prevalent hotspots for atypical mutations were the P-loop (L718–V726, 13.6%) and the C-terminal loop of the αC-helix (A767–G779, 29.4%, Fig. 1c).

To assess the effect of atypical *EGFR* mutations on patient outcomes, we determined the time to treatment failure^18^ (TTF) of patients with NSCLC containing classical or atypical *EGFR* mutations. When treated with an EGFR TKI, patients with atypical *EGFR* mutations had a shorter TTF compared with patients with classical *EGFR* mutations (Fig. 1d, hazard ratio (HR) = 1.8, *P* < 0.0001), even when patients with Ex20ins were excluded from the analysis (Fig. 1e, HR = 1.6, *P* < 0.0001) or when patients were stratified by mutation exon location (Fig. 1e, Extended Data Fig. 1c). When patients were stratified by TKI treatment, those with classical *EGFR* mutations had a longer TTF than those with atypical *EGFR* mutations when treated with first-generation (HR = 1.9, *P* = 0.0005) or third-generation TKIs (HR = 3.0, *P* < 0.0001) (Extended Data Fig. 1d, e). A similar trend was observed for second-generation TKIs; however, the difference was not statistically significant (HR = 1.7, *P* = 0.10) (Extended Data Fig. 1f). Validating these findings in the cBioPortal database, patients with atypical *EGFR* mutations had a shorter progression free interval^19^ and overall survival, irrespective of treatment (Extended Data Fig. 1g, h).

## Structural groups predict drug response

**Fig. 2:**
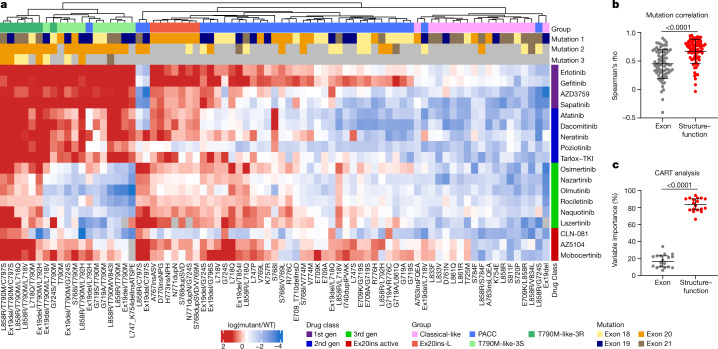
*EGFR* mutations can be separated into four distinct subgroups. **a**, Heat map with unsupervised hierarchical clustering of log(mutant/wild type (WT)) ratios from Ba/F3 cells expressing indicated mutations after drug treatment. To determine the mutant/WT ratio, half-maximal inhibitory concentration (IC_50_) values for each drug and cell line were calculated and then compared to the average IC_50_ values for Ba/F3 cells expressing wild-type *EGFR* (+10 ng ml^−1^ EGF). Squares are representative of the median of *n* = 3 replicates. The order of co-occurring mutations was assigned arbitrarily. Groups were assigned on the basis of structural predictions (Methods). Gen, generation. **b**, Dot plot of Spearman’s rho values for correlations of mutations versus exon-based group averages or structure–function-based averages for each drug. Dots are representative of rho value of each mutation; bars show mean ± s.d., *n* = 77 cell lines or mutations. **c**, Dot plot of variable importance calculated from CART. Dots are representative of variable importance for each drug; bars show median + 95% confidence interval of variable importance for all drugs (*n* = 18 drugs) (Supplementary Table 2). In **b**, **c**, *P* value was determined using a paired two-sided *t*-test. Source data.

**Extended Data Fig 2:**
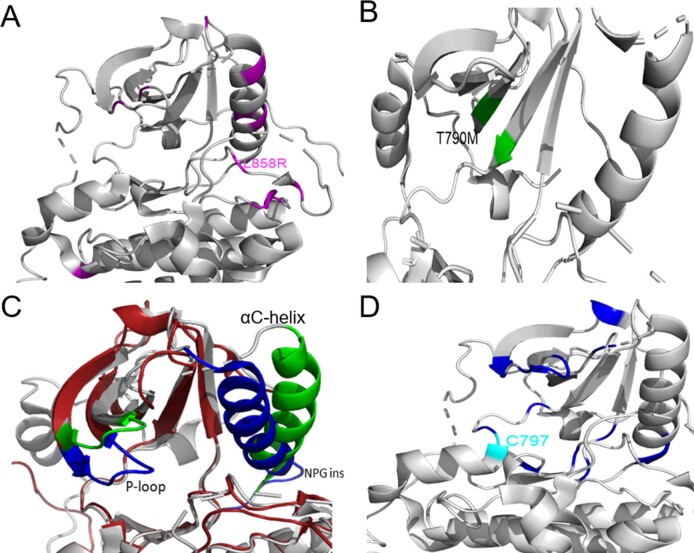
Mutational mapping of EGFR mutations. **a**–**d**, Mutational mapping of classical-like (**a**), T790M-like (**b**), exon 20 loop insertion (red/blue) and WT (grey/green) (**c**), and PACC mutants (**d**) onto WT EGFR crystal (PDB 2ITX).

**Extended Data Fig 3:**
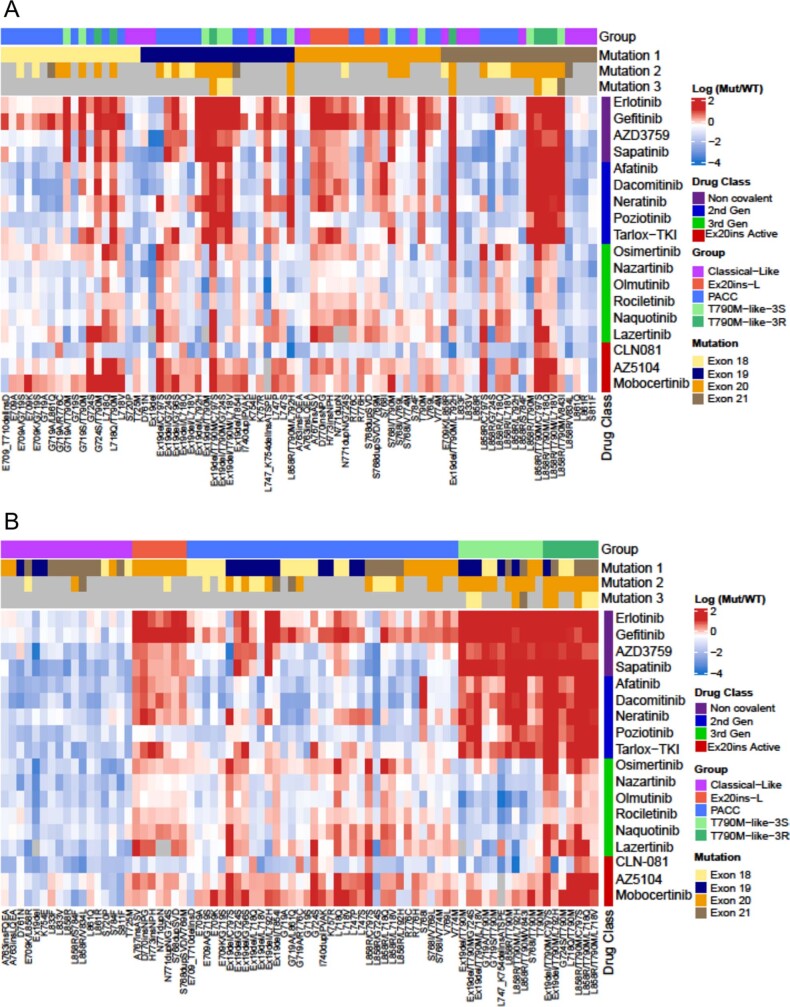
Heat maps generated through supervised clustering by structure-function-based groups cluster drug sensitivity better than exon-based groups. **a**, **b**, Heat maps supervised clustering by exon-based (**a**) or structure-function-based (**b**) groups of log (Mutant/WT) ratios from Ba/F3 cells expressing indicated mutations after 72 h of indicated drug treatment. To determine the mutant/WT ratio, IC_50_ values for each drug and cell line were calculated and then compared to the average IC_50_ values of Ba/F3 cells expressing WT *EGFR* (+10ng/ml EGF to maintain viability). Squares are representative of the median of n=3 replicates. For co-occurring mutations, the order of mutations 1, 2, and 3 were assigned arbitrarily. Groups were assigned based on structural predictions Source data.

**Extended Data Fig. 4:**
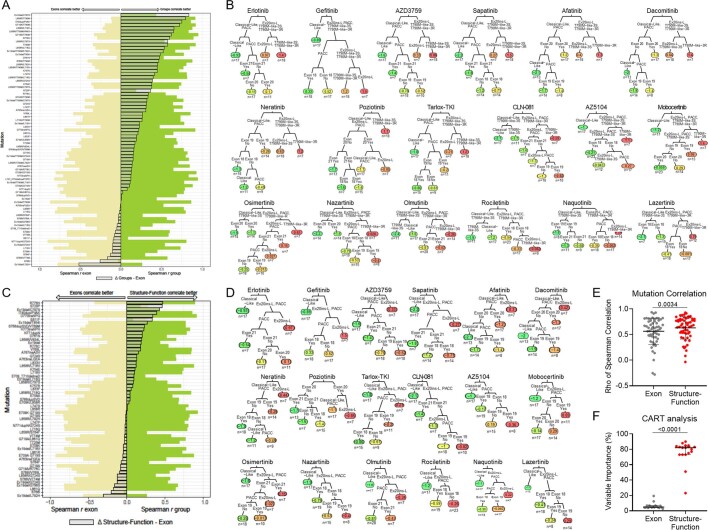
Structure-function-based groupings are more predictive of drug and mutation sensitivity compared to exon-based groupings. **a**, Bar plot of Spearman rho values for indicated mutations compared to exon-based groups (yellow) or structure-function-based groups (green). The delta of the two rho values is shown as an overlapped grey bar. When the delta bar shifts to the right, the spearman rho value was higher for structure-function-based groups, and when the grey bar shifts to the left, the spearman rho value was higher for the exon-based groups. **b**, Representative classification and regression trees for each indicated drug. Colors represent drug sensitivity (green) or resistance (red) as defined by log (mutant IC50/WT EGFR IC50). **c**, Bar plot of Spearman rho values for indicated mutations (excluding T790M mutations) compared to exon-based groups (yellow) or structure-function-based groups (green). The delta of the two rho values is shown as an overlapped grey bar. **d**, Representative classification and regression trees for each indicated drug excluding T790M from the analysis. Colors represent drug sensitivity (green) or resistance (red) as defined by log (mutant IC50/WT EGFR IC50). **e**, Dot plot of rho values from Spearman correlations of mutations vs exon-based group averages or structure-function based averages for each drug excluding T790M mutations. Dots are representative of each mutation; bars are representative of the average rho value ± standard deviation (SD). p-value was determined using a paired two-sided *t*-test, and n = 59 cell lines/mutations. **f**, Dot plot of variable importance calculated as sum of the goodness of split for each split in the classification and regression trees (CART). Dots are representative of variable importance for each drug in the exon and structure-function-based groups as indicated and excluding T790M mutations. Bars are representative of the median + 95% confidence interval of variable importance for all drugs (Supplementary Table 3). p-value was determined using a paired two-sided *t*-test, and n = 18 drugs Source data.

We generated a panel of 76 cell lines expressing *EGFR* mutations spanning exons 18–21 and screened these cell lines against 18 EGFR inhibitors representing first- (non-covalent), second (covalent) and third- (covalent, T790M targeting) generation and Ex20ins-active TKIs (Supplementary Table 1). Using hierarchical clustering of in vitro selectivity over WT EGFR and mutational mapping of *EGFR* mutations, we observed four distinct subgroups of *EGFR* mutations: classical-like mutations that were distant from the ATP-binding pocket (Extended Data Fig. 2a), T790M-like mutations in the hydrophobic core (Extended Data Fig. 2b), insertions in the loop at the C-terminal end of the αC-helix in exon 20 (Ex20ins-L; Extended Data Fig. 2c), and mutations on the interior surface of the ATP-binding pocket or C-terminal end of the αC-helix, which were predicted to be P-loop and αC-helix compressing (PACC) (Fig. 2a, Extended Data Fig. 2d). Supervised heat maps of mutant/wild-type ratios by exon location (Extended Data Fig. 3a) and structure–function groups (Extended Data Fig. 3b) showed distinct differences, suggesting that structure–function-based groups better defined groups of mutations by drug sensitivity than exon-based classification. To test this hypothesis, we calculated the correlations of drug sensitivity and selectivity for each mutation to the predicted drug sensitivity by exon or structure–function groups (Extended Data Fig. 4a) and then compared the median rho value of each correlation for both groups. We found that structure–function-based groups were more predictive of mutation sensitivity than exon-based groups (*P* < 0.0001) (Fig. 2b). We used a secondary approach employing machine learning to analyse data by classification and regression trees (CART) and determine variable importance^20,21^ (Extended Data Fig. 4b). Structure–function-based groups had a higher variable importance than exon-based groups, suggesting that structure–function-based groups were more predictive of which mutational groups would be most sensitive to a particular drug compared with exon-based groups (*P* < 0.0001) (Fig. 2c). We validated these findings without T790M mutations (Extended Data Fig. 4c, d), and structure–function-based groups remained more predictive of mutation and drug sensitivity than exon-based groups (*P* = 0.0034 and *P* < 0.0001, respectively) (Extended Data Fig. 4e, f).

**Extended Data Fig. 5:**
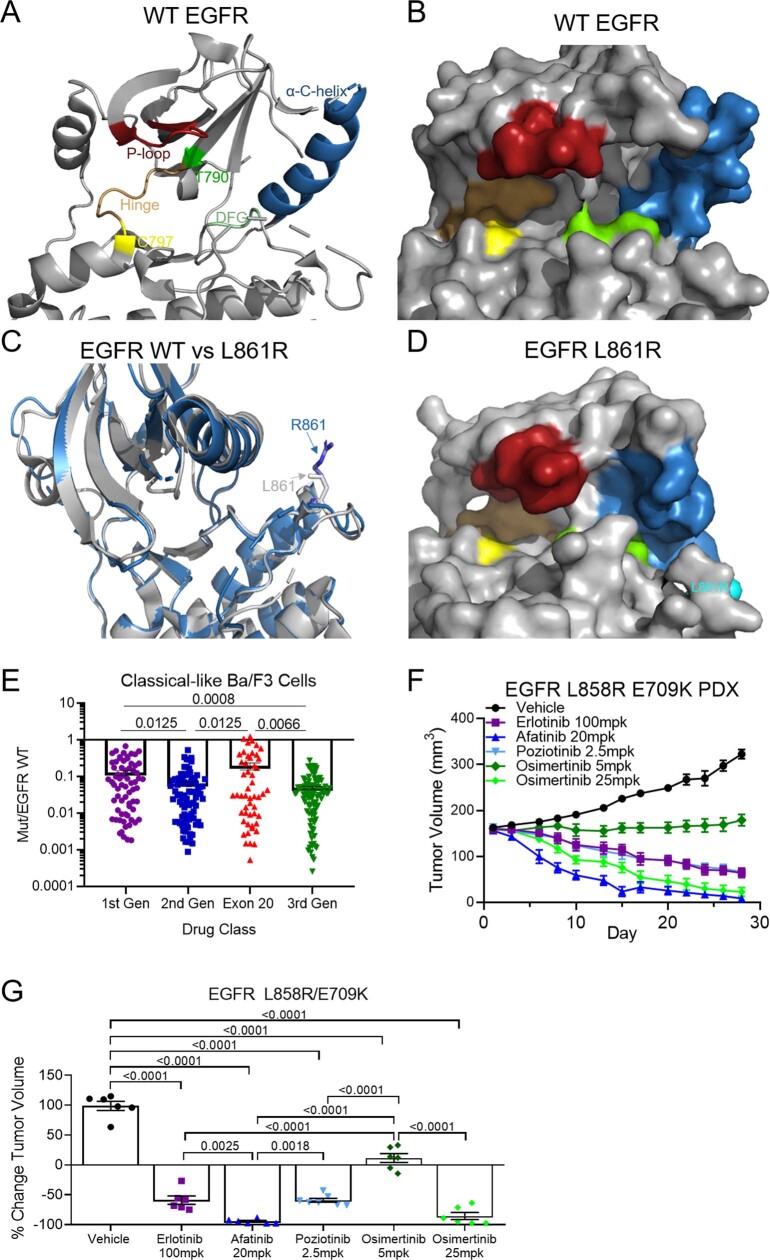
Classical-like EGFR mutations are not predicted to alter the drug-binding pocket and are most sensitive to 3^rd^-gen EGFR TKIs. **a**, **b**, Rendering of crystal structure of WT EGFR (PDB 2ITX) visualized as both a ribbon (**a**) and space filling (**b**) models. Residues important in receptor signaling and drug binding are highlighted. **c**, **d**, Overlapped rendering of WT crystal (grey) (**c**) and L861R (blue) and space filing model (**d**) of L861Q demonstrate the R861 substitution is distal from the drug binding pocket and has minimal impact on the overall structure of EGFR compared to WT. **e**, Dot plot of mutant/WT IC_50_ values of Ba/F3 cells expressing classical-like EGFR mutations and treated with indicated classes of EGFR TKIs. Dots are representative of average of n=3 replicate mutant/WT IC_50_ values of individual cell lines expressing classical-like mutations with individual drugs. Bars are representative of average mutant/WT IC_50_ values ± SEM for each class of EGFR TKI and all classical-like cell lines. p-values were determined by one-way ANOVA with unequal SD as determined by Brown-Forsythe test to determine differences in SD. Holm-Sidak’s multiple comparisons test was used to determine differences between groups. **f**, Tumor growth curves for PDXs harboring EGFR L858R E709K complex mutation treated with indicated inhibitors. Tumors were measured three times per week and symbols are average of tumor volumes ± SEM. Mice were randomized into six groups: vehicle (N=6), poziotinib 2.5mg/kg (N=7), erlotinib 100mg/kg (N=6), afatinib 20mg/kg (N=6), osimertinib 5mg/kg (N=6), and osimertinib 25mg/kg (N=6). Mice received drug 5 days per week, and mice were euthanized at day 28 to harvest tumors. **g**, Dot plot of percent change in tumor volume on day 28 of tumors described in **f**. Dots are representative of each tumor, and bars are representative of average ± SEM for each group. Statistical differences were determined by ordinary one-way ANOVA with post-hoc Tukey’s multiple comparisons test to determined differences between groups vehicle, N=6 mice, poziotinib 2.5mg/kg, N=7 mice, erlotinib 100mg/kg, N=6 mice, afatinib 20mg/kg, N=6 mice, osimertinib 5mg/kg, N=6, and osimertinib 25mg/kg, N=6 mice. Source data

Classical-like, atypical *EGFR* mutations were predicted to have little effect on the overall structure of EGFR compared with wild-type EGFR (Extended Data Fig. 5a–d) and were sensitive and selective for all classes of EGFR TKIs, particularly third-generation TKIs, in vitro and in vivo (Extended Data Fig. 5e–g). Mutations and assigned groups are in Supplementary Table 4.

## Exon 20 mutations are heterogenous

**Extended Data Fig. 6:**
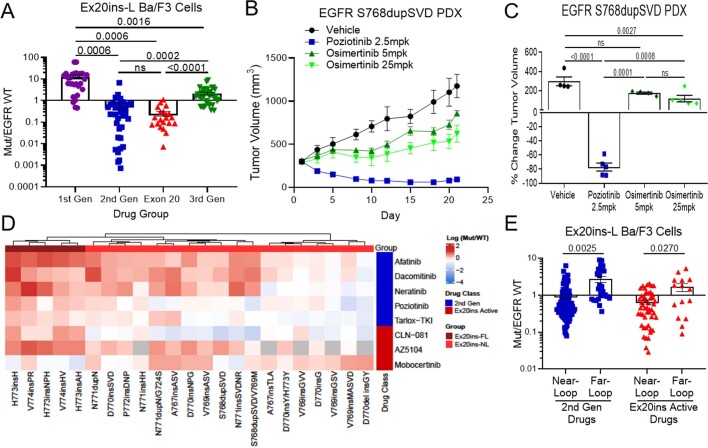
Exon 20 loop insertions are a distinct class of *EGFR* mutations. **a**, Dot plot of mutant/WT IC_50_ values of Ba/F3 cells expressing exon 20 loop insertion mutations and treated with indicated classes of EGFR TKIs. Dots are representative of average of n=3 replicate mutant/WT IC_50_ values of individual cell lines expressing exon 20 insertion mutations with individual drugs. Bars are representative of average mutant/WT IC_50_ values ± SEM for each class of EGFR TKI and all Ba/F3 cell lines. p-values were determined by one-way ANOVA with unequal SD as determined by Brown-Forsythe test to determine differences in SD. Holm-Sidak’s multiple comparisons test was used to determine differences between groups. **b**, Tumor growth curves for PDXs harboring EGFR S768dupSVD exon 20 insertion mutation treated with indicated inhibitors. Tumors were measured three times per week and symbols are average of tumor volumes ± SEM. Mice were randomized into four groups: vehicle (N=4), poziotinib 2.5mg/kg (N=5), osimertinib 5mg/kg (N=4), and osimertinib 25mg/kg (N=5) . Mice received drug 5 days per week, and mice were euthanized at day 21 to harvest tumors. **c**, Dot plot of percent change in tumor volume on day 21 of tumors described in panel **c** (vehicle, N=4 mice, poziotinib 2.5mg/kg, N=5 mice, osimertinib 5mg/kg, N=4 mice, and osimertinib 25mg/kg, N=5 mice). Dots are representative of each tumor, and bars are representative of average ± SEM for each group. Statistical differences were determined by ordinary one-way ANOVA with post-hoc Tukey’s multiple comparisons test to determined differences between groups. **d**, Heat map with unsupervised hierarchical clustering of log (Mutant/WT) ratios from Ba/F3 cells expressing indicated exon 20 loop insertions after 72 h of indicated drug treatment. Squares are representative of the median of n=3 replicates. **e**, Bar plot of average mutant/WT ratio of Ba/F3 cells expressing exon 20 loop insertions separated by mutational groups for indicated drug classes. Bars are representative of average mutant/WT ratio for all mutations and drugs in the indicated groups, dots are representative of average (n=3) mutant/WT ratio of individual mutations and drugs. Error bars are representative of SEM for each bar. Statistical differences between near- and far-loop mutants was determined by two-sided unpaired *t*-test with unequal variance Source data.

Studies showed that exon 20 mutations are heterogenous in their response to TKIs^10,22^. Insertions in the αC-helix (for example, an FQEA insertion at A763 (A763insFQEA)) were pan-sensitive to EGFR TKIs^23,24^, whereas those in the loop following the αC-helix (A767–C775) were not^25,26^, and the T790M mutation was sensitive to third- but not first- or second-generation TKIs. We found that most exon 20 point mutations were PACC mutations; that exon 20 insertions in the αC-helix were classical-like mutations; and, that the remainder of exon 20 insertions occurring in the C-terminal loop of the αC-helix were a distinct subgroup: exon 20 loop insertions (Ex20ins-L) (Fig. 2a). Ex20ins-L were sensitive only to select second-generation TKIs (that is, poziotinib and tarlox-TKI) and Ex20ins-active TKIs in vitro and in vivo (Fig. 2a, Extended Data Fig. 6a–c). However, even within Ex20ins-L mutations, some degree of heterogeneity in drug sensitivity was observed (Fig. 2a, Extended Data Fig. 6a). Using a panel of an additional 15 Ba/F3 cell lines expressing Ex20ins-L mutations spanning A767–V774, we found that Ex20ins-L mutations could be subdivided into two subgroups: near- and far-loop Ex20ins (Extended Data Fig. 6d). Exon 20 near-loop insertions (Ex20ins-NL) were more sensitive to second-generation and Ex20ins-active TKIs compared with exon 20 far-loop insertions (Ex20ins-FL) (*P* = 0.0025 and *P* = 0.027, respectively) (Extended Data Fig. 6e). These data exemplify that mutations within an exon are heterogenous and that an exon-based classification is unlikely to be optimal for guiding treatment decisions.

## Drug repurposing for resistant mutations

**Extended Data Fig. 7:**
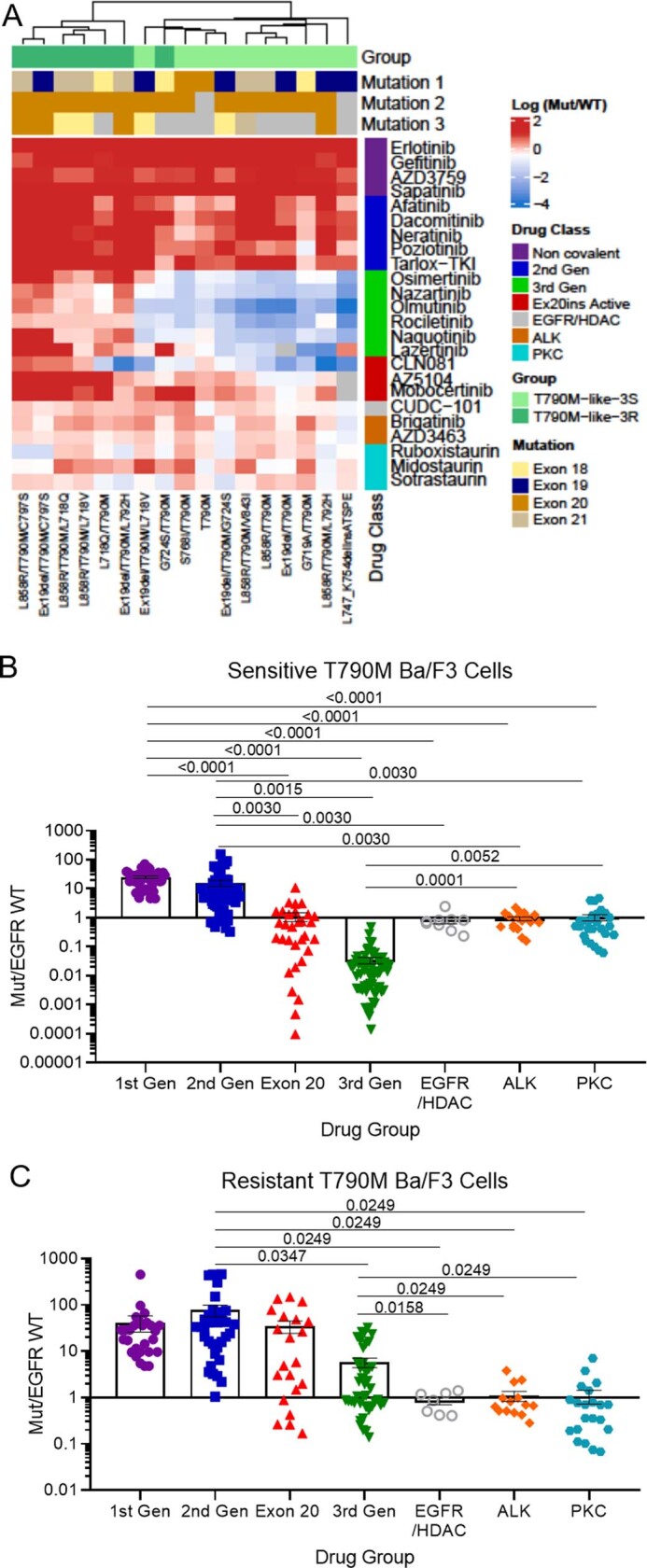
Drug repurposing can overcome T790M-like resistance mutations. **a**, Heat map with unsupervised hierarchical clustering of log (mutant/WT) ratios from Ba/F3 cells expressing indicated mutations after 72 h of indicated drug treatment. Squares are representative of the median of n=3 replicates. For co-occurring mutations, the order of mutations 1, 2, and 3 were assigned arbitrarily. Groups were assigned based on hierarchal clustering and known resistance mutations. **b**, **c**, Dot plot of mutant/WT IC_50_ values of Ba/F3 cells expressing T790M-like-3S (3^rd^-gen TKI sensitive) (**b**) and T790M-like-3R (3^rd^-gen TKI resistant) (**c**) treated with indicated classes of EGFR TKIs. Dots are representative of average of n=3 replicate mutant/WT IC_50_ values of individual cell lines with individual drugs and drugs of each class are grouped together. Bars are representative of average mutant/WT IC_50_ values ± SEM for each class of EGFR TKI and all cell lines. p-values were determined by one-way ANOVA with unequal variance as determined by Brown-Forsythe test to determine differences in variance. Holm-Sidak’s multiple comparisons test was used to determine differences between groups Source data.

Although all T790M-like mutants had at least one mutation in the hydrophobic core, there were two distinct subgroups of T790M-like mutants—third-generation TKI sensitive (T790M-like-3S) and third-generation TKI resistant (T790M-like-3R) (Extended Data Fig. 7a). Previous reports have shown that protein kinase C^27^ (PKC) and anaplastic lymphoma kinase^28,29^ (ALK) inhibitors exhibit off-target activity for EGFR mutations including T790M, and the non-covalent nature of these compounds predict that they retain activity in mutations that interrupt covalent binding. T790M-like-3S mutants had high selectivity for third-generation TKIs and some Ex20ins-active inhibitors and moderate selectivity for ALK and PKC inhibitors (Extended Data Fig. 7b). T790M-like-3R mutants, complex mutations comprising T790M and a known drug-resistance mutation (that is, C797S^30^, L718X^31^ or L792H^18,31^), were resistant to classical EGFR TKIs but retained selectivity for select ALK and PKC inhibitors such as brigatinib or midostaurin (Extended Data Fig. 7c). These data support expanding testing of ALK and/or PKC inhibitors or development of novel non-covalent inhibitors for the broader group of T790M-like-3R mutations.

## Second-generation TKIs inhibit PACC mutations

**Fig. 3:**
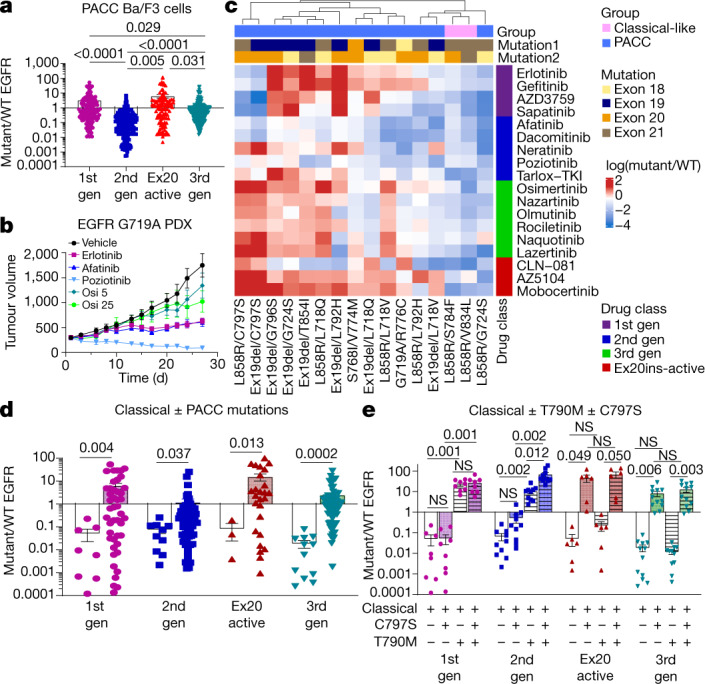
PACC mutations are robustly sensitive to second-gneeration TKIs. **a**, Dot plot of mutant/WT IC_50_ values of Ba/F3 cells expressing PACC mutations. **b**, Tumour growth curves for PDXs containing EGFR G719A PACC mutation treated with TKIs five days per week. Symbols show mean tumour volume ± s.e.m., *n* = 5 mice. Osi 5, osimertinib 5 mg kg^−1^; osi 25, osimertinib 25 mg kg^−1^. **c**, Heat map with unsupervised hierarchical clustering of log(mutant/WT) ratios from Ba/F3 cells expressing indicated mutations after drug treatment. Squares represent the median of *n* = 3 replicates. Mutation order was assigned arbitrarily; groups were assigned on the basis of predicted mutational impact. **d**, Dot plot of mutant/WT IC_50_ values of Ba/F3 cells expressing classical EGFR mutations (white bars) with or without PACC mutations (coloured bars). In **a**, **d**, *P* values were determined by one-way analysis of variance (ANOVA) with unequal s.d. and Holm–Sidak’s multiple comparisons test. **e**, Average mutant/WT ratio of Ba/F3 cells expressing classical EGFR mutations (white bars), and classical EGFR mutations plus C797S (shaded bar), T790M (hashed bars) or T790M and C797S (shaded and hashed bars). *P* values were determined by one-way ANOVA with repeated measures and post hoc Fishers’ multiple comparisons test. In **a**, **f**, **h**, bars show mean ± s.e.m. of mutant/WT ratio for all mutations and drugs; dots show representative average mutant/WT of *n* = 3 replicates. Source data.

**Extended Data Fig. 8:**
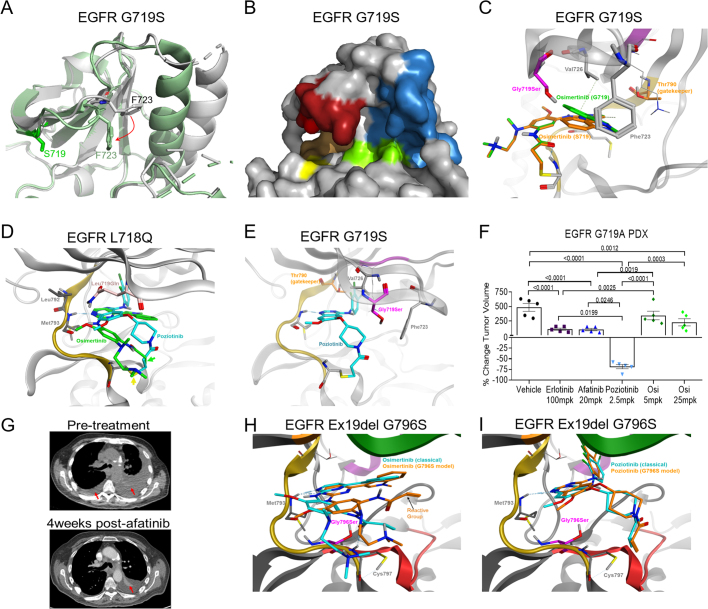
PACC mutations alter the orientation of the P-loop and/or αC-helix and are sensitive to 2^nd^-gen TKIs. **a**, Overlap of G719S (PDB 2ITN, green) and WT EGFR (PDB 2ITX, grey) crystal structures demonstrate arobust shift of F723 (red arrow) in the P-loop orienting the benzyl ring in a downward position condensing the P-loop in the drug binding pocket. Further, G719S displays an inward shift of the αC-helix compared to the WT crystal structure. **b**, Surface representation of G719S (PDB 2ITN) with P-loop (red), αC-helix (blue), hinge region (orange), C797 (yellow), and DFG motif (green) highlighted to demonstrate steric hindrance of drug binding pocket caused by shifted P-loop. **c**, Comparison of osimertinib bound to wild-type EGFR (PDB 4ZAU, green) or EGFR G719S (PDB 2ITN, purple) demonstrates destabilization of TKI-protein interactions. **d**, In silico homology model of EGFR L718Q (pink) with predicted binding modes of osimertinib and poziotinib structures demonstrates that Q718 hinders the interaction of osimertinib (green) with M793 and shifts the Michael acceptor (reactive group, green arrow) out of alignment with C797 (yellow arrow). In contrast, poziotinib (blue) is less effected by Q718 and is still positioned to react with C797, even in the context of L718Q mutations. **e**, In silico modeling of EGFR G719S (purple) with poziotinib (blue) shows no predicted changes in poziotinib binding or TKI-protein interactions. **f**, Dot plot of percent change in tumor volume on day 28 of tumors described in Fig. 3c. Dots are representative of each tumor, and bars are representative of average ± SEM for each group, N=5 mice per treatment group. Statistical differences were determined by ordinary one-way ANOVA with post-hoc Tukey’s multiple comparisons test to determined differences between groups. **g**, Representative CT images from a patient harbouring E709K G719S complex mutation before and after four weeks of afatinib treatment. Red arrow indicates resolved pleural effusion on the left lobe and reduced pleural effusion and tumor in the right lobe. **h**, In silico modeling of EGFR Ex19del G796S (purple) with osimertinib in the reactive (blue) and predicted (orange) conformations demonstrate destabilization of TKI-protein interactions in the hinge region (yellow), displacing the reactive group (arrow). **i**, In silico modeling of EGFR Ex19del G796S (purple) with the reactive conformation of poziotinib (blue) and the predicted conformation of poziotinib (orange) predicted minimal changes in poziotinib binding and similar TKI-protein interactions. Source data

PACC mutations comprise mutations spanning exons 18–21 including G719X, L747X, S768I, L792X and T854I and were predicted to alter the orientation of the P-loop or αC-helix (Extended Data Fig. 8a, b). In silico analysis of the interaction of osimertinib with PACC mutations G719S and L718Q predicted that changes in the orientation of the P-loop alter the position of TKI stabilization points tilting the indole ring of osimertinib away from the P-loop, destabilizing drug binding (Extended Data Fig. 8c, d). By contrast, second-generation TKIs do not interact with the P-loop of EGFR and maintain interaction points in the hydrophobic cleft (Extended Data Fig. 8d, e). When we compared the selectivity of EGFR TKIs for PACC mutations, we found that second-generation TKIs were significantly more selective for PACC mutations than any other TKI class (Fig. 3a). In vivo, we also observed that NSCLC patient-derived xenografts (PDXs) containing G719A mutations were resistant to the third-generation TKI osimertinib, but most sensitive to the second-generation TKI poziotinib (Fig. 3b, Extended Data Fig. 8f). Notably, a patient with a complex PACC mutation, E709K/G719S, saw clinical benefit and tumour shrinkage with afatinib treatment after progressing on osimertinib (Extended Data Fig. 8g).

**Extended Data Fig. 9:**
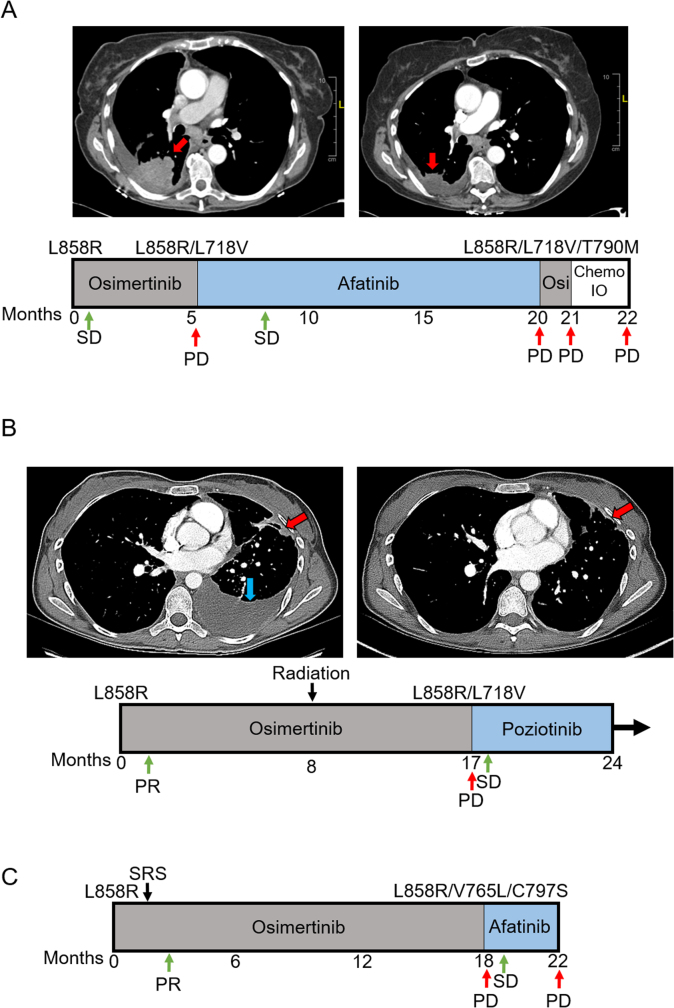
2^nd^-gen TKIs confer durable clinical benefit in patients with acquired osimertinib-resistant NSCLC. **a**, Representative CT images from a patient after 5.5 months  of osimertinib and 6 months after afatinib treatment. Red arrow indicates lesion harboring L858R/L718V. **b**, CT scan of a patient after 17 months of osimertinib treatment showed new pleural lesion that tested positive for both EGFR L858R and L718V mutations (red arrow), and CT image of patient four weeks after beginning poziotinib treatment shows reduction in size of the pleural lesion (red arrow). Blue arrow indicates resolved pleural effusion. **a**, **b**, Schematic below CT images shows timeline of patient treatments and outcomes. **c**, Schematic representations of a patient treatments and outcomes that acquired two PACC mutations (V765L and C797S) after 18 months of osimertinib treatment. PR = partial response, PD = progressive disease, SD = stable disease, SRS = stereotactic radiosurgery Chemo/IO = carboplatin/ pemetrexed+ pembrolizumab.

Similarly, acquired PACC mutations co-occurring with primary classical *EGFR* mutations retained sensitivity to second-generation TKIs while acquiring resistance to third-generation TKIs in an allele-specific manner (Fig. 3c, d). In silico analysis of acquired PACC mutation, G796S, co-occurring with Ex19del was predicted to confer resistance to third-generation TKIs such as osimertinib by shifting the hinge region of the receptor, preventing stabilization of osimertinib at M793 and displacing the acrylamide group of osimertinib away from C797 (Extended Data Fig. 8h). However, second-generation TKIs were less affected by shifts in the hinge region of the receptor and were predicted to maintain the orientation of the acrylamide group (Extended Data Fig. 8f). Previous studies have reported that C797S mutations confer resistance to third-generation TKIs even without the presence of T790M^30^ (Fig. 3e). Similarly, C797S mutations without T790M conferred resistance to Ex20ins-active inhibitors, but not first- or second-generation TKIs unless T790M was present (Fig. 3e). Retrospectively, we identified three patients with NSCLC containing *EGFR* L858R mutations that received first-line osimertinib and subsequently developed an *EGFR*-dependent mechanism of resistance. In all patients, a PACC mutation was identified upon biopsy at progression (Extended Data Fig. 9a–c). Two patients acquired a L718V mutation, and one acquired two PACC mutations (V765L and C797S). All patients were treated with a second-generation TKI and experienced clinical benefits of stable disease and tumour shrinkage (Extended Data Fig. 9a–c). These data demonstrate that both primary and acquired PACC mutations are sensitive to second-generation TKIs, and structure–function-based groupings could identify a novel class of mutations, PACC mutations, for which second-generation TKIs had higher selectivity and efficacy than third-generation drugs.

## Structure-based groups predict outcomes

**Fig. 4:**
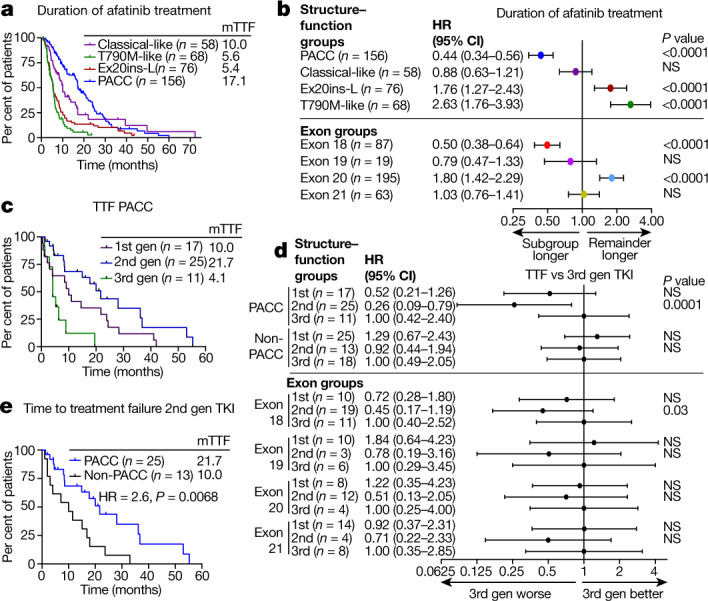
Structure–function groups better predict patient outcomes than exon-based groups. **a**, Kaplan–Meier plot of DOT of patients with NSCLC tumours containing atypical *EGFR* mutations (*n* = 358 patients) stratified by structure-based groups treated with afatinib. **b**, Forest plot of HRs calculated from Kaplan–Meier plots in **a** and Extended Data Fig. 10c. In **a**, **b**, classical-like, *n* = 58; T790M-like, *n* = 68; Ex20ins-L, *n* = 76; PACC, *n* = 156; exon 18, *n* = 87; exon 19, *n* = 19; exon 20, *n* = 195; exon 21, *n* = 63. **c**, Kaplan–Meier plot of TTF of patients with PACC mutations treated with first-, second- or third-generation EGFR TKIs. **d**, Forest plot of HRs calculated from Kaplan–Meier plots in **c** and Extended Data Fig. 10d–h. In **c**, **d**, PACC, *n* = 53; non-PACC, *n* = 56; exon 18, *n* = 40; exon 19, *n* = 19; exon 20, *n* = 24; exon 21, *n* = 26. **e**, Kaplan–Meier plot of TTF of patients with PACC mutations (*n* = 25) or non-PACC mutations (*n* = 13) treated with second-generation TKIs. In **a**–**e**, HRs and *P* values were calculated using two-sided Mantel–Cox log-rank tests. In **b**, **d**, data are representative of HR ± 95% confidence interval. Source data.

**Extended Data Fig. 10:**
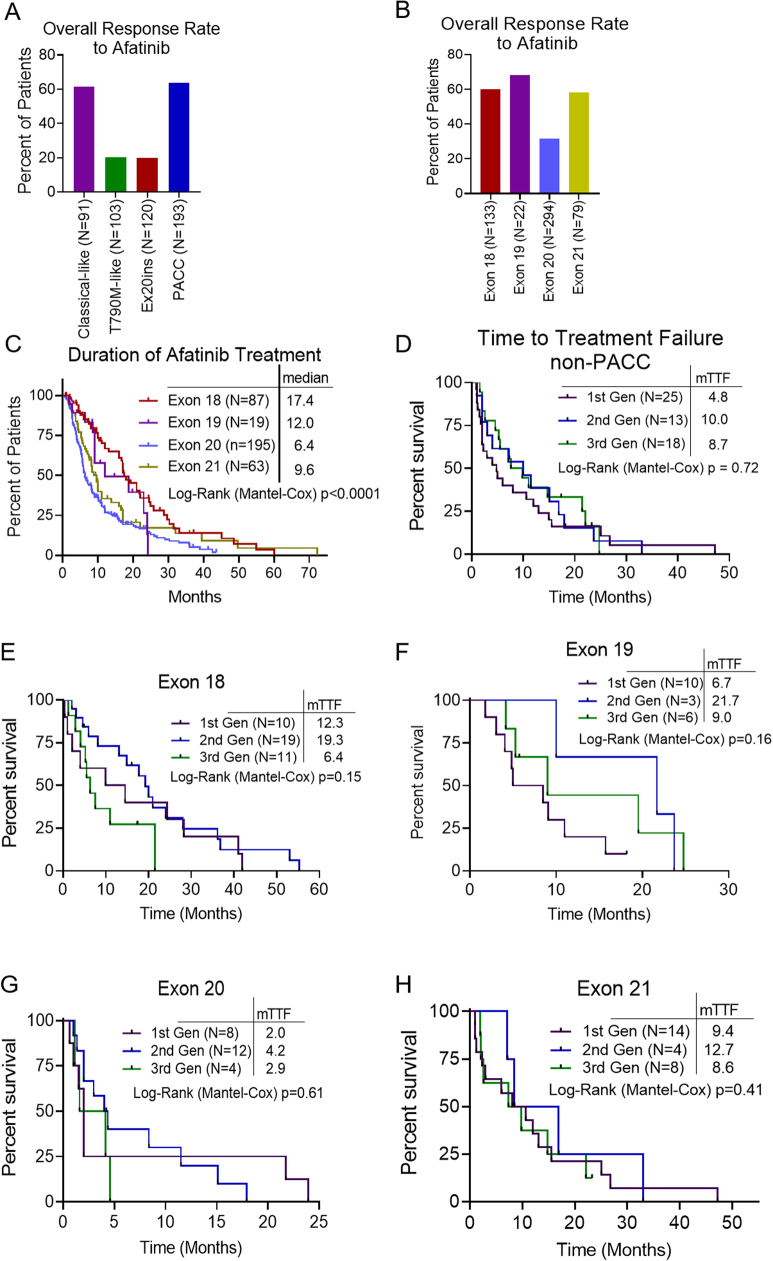
Structure-function groups identify patients with greater benefit to 2^nd^-gen TKIs than exon-based groups. **a**, **b**, Overall response rate to afatinib stratified by structure-function-based groups (N= 507: Classical-like N=91, T790M-like N=103, Ex20ins-L N=120, and PACC N=193) (**a**) or exon based groups (N= 528: Exon 18 N=133, Exon 19 N=22, Exon 20 N=294, Exon 21 N=79) (**b**). When mutations were not explicitly stated (N=21), those patients were excluded from the structure-function based analysis. **c**, Kaplan-Meier plot of duration of afatinib treatment of patients with NSCLC tumors harboring atypical *EGFR* mutations (N= 364 patients) stratified by exon-based groups. Exon 18 N=87, Exon 19 N=19, Exon 20 N=195, and Exon 21 N=63. **d**, Kaplan-Meier plot of TTF of patients with NSCLC harboring non-PACC atypical EGFR mutations (N= 56) treated with 1^st^- (N=25), 2^nd^- (N=13), or 3^rd^-gen (N=18) EGFR TKIs. Forrest plot comparing PACC and non-PACC mutants can be found in Fig. 4d. **e**–**h**, Kaplan-Meier plots of TTF of patients atypical EGFR mutations stratified by EGFR TKI class for exons 18 (N=40) (**e**), 19 (N=19) (**f**), 20 (N=24) (**g**), and 21 (N=26) (**h**). Forrest plot comparing HRs and p-values across exons can be found in Fig. 4d. Source data

To determine whether structure–function-based groups could identify patients who are most likely to benefit from a treatment better than exon-based groups, we used a publicly available database of outcomes for patients harbouring atypical EGFR mutations treated with afatinib^32^ and determined overall response rate (ORR) and duration of treatment (DOT). Structure–function-based grouping showed clear differences between sensitive (classical-like and PACC) and resistant (T790M-like and Ex20ins-L) subgroups (ORR of 63% versus 20%), whereas exon-based groups had less variation between groups (Extended Data Fig. 10a, b). Structure–function-based groups identified that patients with PACC mutations (*n* = 156) had a significantly longer DOT for afatinib than other structure-based groups (DOT: 17.1 months, *P* < 0.0001) (Fig. 4a, b). Using exon-based groups, we also identified that patients with exon 18 mutations (*n* = 87) had a longer DOT than patients with mutations in exons 19–21 (DOT: 17.4 months, *P* < 0.0001) (Fig 4b, Extended Data Fig. 10c); however, the structure-based approach identified nearly twice as many individuals who benefited from afatinib treatment.

To determine whether structure-based groups could identify which class of inhibitors would provide the most benefit to patients with atypical EGFR mutations, we performed retrospective analyses of TTF of patients with atypical *EGFR* mutations treated with EGFR TKIs in MD Anderson Cancer Center GEMINI and Moffitt Cancer Center databases, and TTF was determined for the first EGFR TKI for which patients were treated. Most patients (80%) were stage IV at diagnosis, and there were no statistical differences in patient characteristics (Supplementary Tables 5, 6). When stratified by structure–function-based groups, patients with PACC mutations treated with second-generation TKIs had a significantly longer TTF than patients treated with either first- or third-generation TKIs (21.7 mo﻿nths versus 10.0 mo﻿nths and 4.1 mo﻿nths, respectively; *P* < 0.0001, HR = 0.23) (Fig. 4c, d). By contrast, TTF was not significantly different between classes of EGFR TKIs in patients with non-PACC mutations (Fig. 4d, Extended Data Fig. 10d). Further, patients with PACC mutations had a longer TTF than patients with non-PACC mutations when treated with second-generation TKIs (21.7 mo﻿nths versus 10.0 mo﻿nths, respectively; HR = 2.6, *P* = 0.0068) (Fig. 4e). When patients were stratified by exon and TTF was calculated for first-, second- and third-generation TKIs, significant differences were observed only in patients with exon 18 mutations treated with second-generation TKIs compared with third-generation TKIs (20.9 mo﻿nths versus 5.5 mo﻿nths; *P* = 0.001, HR = 0.29) (Fig 4d, Extended Data Fig. 10e–h). Therefore, structure–function classification identified not only a larger subgroup of patients, but also a subgroup with greater benefit from second-generation TKIs than the exon-based classification.

## Discussion

**Fig. 5:**
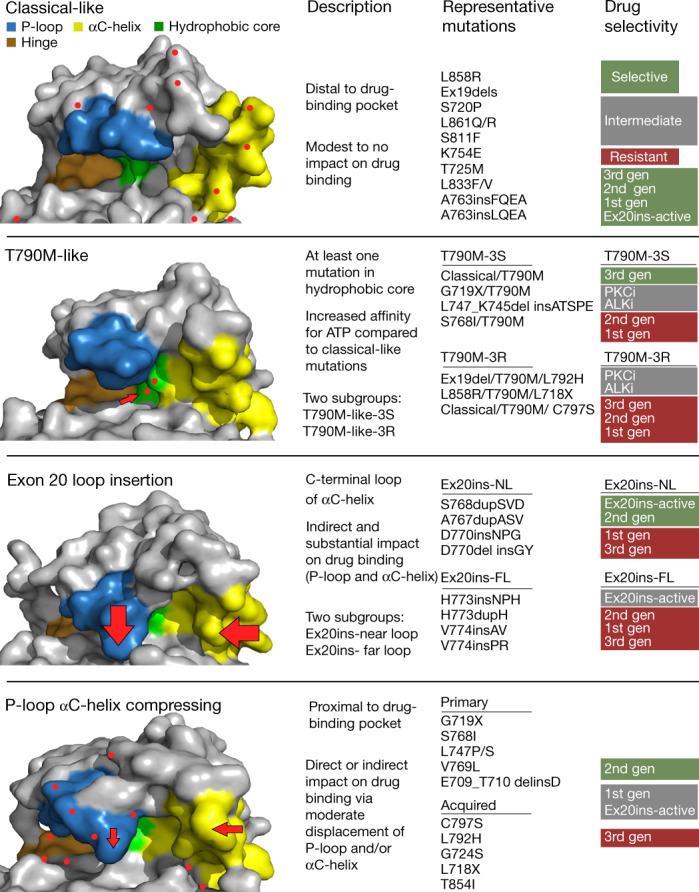
EGFR mutations can be divided into four distinct subgroups. Representative space-filling models of each EGFR subgroup demonstrate changes in overall shape of drug-binding pocket. The P-loop (blue), hinge region (ATP-binding site (orange)), hydrophobic cleft (green), and αC-helix (yellow) are shown. Red dots represent location of mutations. Arrows indicate location of structural changes compared with wild-type EGFR. The most common mutations are shown for each group, and drug sensitivity or selectivity is colour-coded and listed from most selective or sensitive (green) to resistant (red). PKCi, PKC inhibitor; ALKi, ALK inhibitor.

The diversity and higher than previously appreciated prevalence of atypical *EGFR* mutations shown here highlights the necessity of comprehensive next-generation sequencing (NGS) for patients with NSCLC. We show that *EGFR* mutations, including atypical mutations, can be divided into four distinct subgroups based on structure and function (Fig. 5), and that structure–function-based groups can predict drug sensitivity and patient outcomes better than exon-based groups. While previous studies have shown activity of second-generation TKIs in patients with specific exon 18 mutations^33,34^, structure–function-based grouping identified a larger subgroup of *EGFR* mutations, PACC mutants, for which second-generation TKIs were more selective than third-generation TKIs. Clinically, second-generation TKIs have been associated with inhibition of wild-type EGFR and related adverse events^15,35,36^; however, most second-generation TKIs are dosed at the maximum tolerated doses, resulting in plasma concentrations 10–100 fold greater than concentrations necessary for inhibiting PACC mutations. Unlike osimertinib, second-generation TKIs have limited activity in the central nervous system, demonstrating the need for novel TKIs with reduced inhibition of wild-type EGFR inhibition and CNS activity that can inhibit PACC mutants.

These findings demonstrate that structure–function-based groups can identify classes of drugs that may be effective for whole groups of mutations, reflecting the observation that mutations in different regions of the gene may induce similar changes in protein structure. For example, L718Q, S768I, and T854I correspond to exons 18, 20, and 21, respectively, but are all PACC mutations with similar structural effects on drug binding. Conversely, mutations within the same exon may induce quite disparate changes. L747_K754del-insATSPE, L747P and E746-A750del mutations occur in exon 19 but are T790M-like, PACC, and classical mutations, respectively, with distinct drug sensitivity and structural effects. A clinical challenge for physicians treating patients with *EGFR*-mutant cancers is to appropriately identify and match patient mutations with the best EGFR TKI. While a separate classification for each individual mutation could provide more precision than the groups described here, validating the clinical activities of different drugs for each mutation is not feasible. The classification presented here provides a framework through which clinicians, informed by internet-based tools or companies providing NGS reports, could more effectively personalize EGFR TKI therapy. Finally, these findings support the notion that for cancers containing oncogenes with diverse mutations, adopting a structure–function-based approach may improve clinical trial design and drug development.

## Methods

No sample size calculations were done to predetermine group sizes, and investigators were not blinded during randomization and outcome assessments.

### Analysis of EGFR variants in MD Anderson Cancer Center GEMINI, Foundation Medicine, Guardant Health and cBioPortal databases

To analyse the numbers and frequencies of different *EGFR* mutations among patients with NSCLC in the MD Anderson Cancer Center GEMINI database, the database was queried for patients with *EGFR* mutations (*n* = 1,054) and manually curated as classical or atypical EGFR mutations. The MD Anderson Cancer Center GEMINI database is prospectively collected from patients consented and enrolled on protocol number PA13-0589 in accordance with the MD Anderson Institutional Review Board.

EGFR mutations were determined from formalin-fixed paraffin-embedded tumours or digital-droplet PCR for blood samples by CLIA-certified methods as previously described^18,37^. In brief, samples from MD Anderson Cancer Center were collected through molecular pathology and mutations were determined by next-generation sequencing panels of tumour tissue DNA (MD Anderson Cancer Center Molecular Diagnostics Laboratory). MD Anderson Molecular Diagnostics Laboratory is a tissue molecular profiling method for NGS-based analysis to detect mutations in hotspot regions of 50 genes, and in April 2016, it was expanded to analyse 134 unique genes for the detection of somatic mutations in coding sequences of 128 genes and selected copy number variations (amplifications) in 49 genes. Moffitt Cancer Center used diagnostic methods such as Clarinet (bi-directional sequencing of exons 18–21 of *EGFR*), Pyrosequencing of *EGFR* gene (exons 18–21), and Moffitt Illumina TruSight Tumor 26 (TST26). Moffitt Trusight is a NGS Illumina sequencing platform with a panel of 170 genes. Commercial NGS platforms including FoundationOne and Guardant360 were used by both MD Anderson and Moffitt Cancer Center as described below.

To identify patients with *EGFR* mutations in the Foundation Medicine database, patient samples taken between November 2011 and May 2020 previously subjected to hybrid-capture based comprehensive genomic profiling using formalin-fixed paraffin-embedded tissue or plasma using previously validated assays^38,39^, were analysed for *EGFR* mutations (*n* = 10,221). Patients were stratified by *EGFR* mutation, and *EGFR* mutations were manually curated as atypical or classical *EGFR* mutations. Classical *EGFR* mutations were defined as L858R point mutations, T790M mutations, and various exon 19 deletions including any deletion in exon 19 beginning at amino acid E746 or L747 and ending at amino acid A755. Deletions also including insertions were allowed and still considered classical exon 19 deletions. Atypical *EGFR* mutations were defined as non-synonymous mutations that were not defined as classical mutations. Patients with *EGFR* mutations where the sequence of the mutation was unknown were excluded from the analysis.

To determine the frequency of individual *EGFR* variants reported across the MD Anderson GEMINI database, cBioPortal, Foundation Medicine and the Guardant Health database, each database was analysed separately, and the average of all databases was determined. To determine the frequency of atypical mutations in the MD Anderson GEMINI and Foundation Medicine databases, atypical mutations were identified as described above and total number of known EGFR mutations across all patients was tabulated. For the analysis of cBioPortal, all non-overlapping studies were selected and exported. For overlapping studies, only the largest dataset was used, and all known EGFR mutations were tabulated. To determine the frequencies of *EGFR* variants from Guardant Health, a database of sequenced circulating free DNA (cfDNA), the Guardant360 clinical database was searched for NSCLC samples tested between November 2016 and November 2019 harbouring *EGFR* mutations (*n* = 5,026 patients). Guardant360 is a CLIA-certified, CAP/NYSDOH accredited comprehensive cfDNA NGS test that reports on SNVs, indels, fusions and SNVs in up to 73 genes. The Guardant360 clinical database, and the four datasets reported here, are enriched in North American patients with NSCLC; the frequency of atypical EGFR mutations may differ in Asia or other regions.

### Analysis of TTF in MD Anderson Cancer Center GEMINI and Moffitt Cancer Center

To determine TTF after EGFR TKI treatment, patients with NSCLC harbouring an EGFR mutation in the tyrosine kinase domain (exons 18–22) were identified in the MD Anderson GEMINI and Moffitt Cancer Center databases. Data collection for Moffitt Cancer Center (MCC) patients was performed under the protocol (MCC 19161), which was formally reviewed and granted approval by MCC in accordance with the Declaration of Helsinki and the 21st Century Cures Act. Outcomes were recorded for patients for only first EGFR TKI. Patients were stratified by classical (L858R or Ex19del, as defined above) or atypical (non-classical). There were 333 patients with NSCLC identified in the MD Anderson GEMINI database who had tumours expressing atypical mutations. Of these patients, 88 patients received at least one line of EGFR TKI treatment. In addition, at Moffitt Cancer Center, there were 21 patients with NSCLC with tumours harbouring atypical EGFR mutations. Clinical parameters were extracted from the respective databases. Patients previously receiving chemotherapy were included, and TTF was calculated for the first EGFR TKI received. TTF was determined as previously described^18^ and defined as time from commencement of EGFR TKI to radiologic progression, TKI discontinuation, or death, and was not based on RECIST criteria. For patients treated beyond progression, radiologic progression was recorded as the end point, and data cut-off was May 2021. Median TTF was calculated using the Kaplan–Meier method. HR and *P* values were determined using GraphPad Prism software and two-sided Mantel–Cox log-rank tests.

### Analysis of OS and PFI from cBioPortal Database

For overall survival (OS) and progression-free interval (PFI), analysis of patients in cBioportal was determined as previously described^19^ for patients receiving any treatment with survival information and qualifying EGFR mutation. This information was curated from cBioportal by selecting all non-overlapping studies of NSCLC. For overlapping studies, the largest database was selected. PFI and OS analysis  were restricted to the tyrosine kinase domain. Median OS and median PFI were calculated using the Kaplan–Meier method. HR and *P* values were determined using GraphPad Prism software and two-sided Mantel–Cox log-rank tests.

### Ba/F3 cell generation, drug screening and IC_50_ approximations

Ba/F3 cells were obtained as a gift from G. Mills (MD Anderson Cancer Center) and maintained in RPMI (Sigma) containing 10% FBS, 1% penicillin-streptomycin and 10 ng ml^−1^ recombinant mIL-3 (R&D Biosystems). To establish stable Ba/F3 cell lines, Ba/F3 cells were transduced with retroviruses containing mutant EGFR plasmids for 12–24 h. Retroviruses were generated using Lipofectamine 2000 (Invitrogen) transfections of Phoenix 293T-ampho cells (Orbigen) with pBabe-Puro based vectors listed in Supplementary Table 7. Vectors were generated by GeneScript or Bioinnovatise using parental vectors from Addgene listed in Supplementary Table 7. After 48–72 h of transduction, 2 µg ml^−1^ puromycin (Invitrogen) was added to Ba/F3 cell lines in complete RPMI. To select for EGFR-positive cell lines, cells were stained with PE-EGFR (Biolegend) and sorted by fluorescence-activated cell sorting. After sorting, EGFR-positive cells were maintained in RPMI containing 10% FBS, 1% penicillin-streptomycin, and 1 ng ml^−1^ EGF to support cell viability. Drug screening was performed as previously described^22,36^. Shortly, cells were plated in 384-well plates (Greiner Bio-One) at 2,000–3,000 cells per well in technical triplicate. Seven different concentrations of TKIs or DMSO vehicle were added to reach a final volume of 40 µl per well. After 72 h, 11 µl of Cell Titer Glo (Promega) was added to each well. Plates were incubated for a minimum of 10 min, and bioluminescence was determined using a FLUOstar OPTIMA plate reader (BMG LABTECH). Raw bioluminescence values were normalized to DMSO control-treated cells, and values were plotted in GraphPad Prism. Non-linear regressions were used to fit the normalized data with a variable slope, and IC_50_ values were determined by GraphPad prism by interpolation of concentrations at 50% inhibition. Drug screens were performed in technical triplicate on each plate and either duplicate or triplicate biological replicates. Mutant to WT ratios for each drug were calculated by dividing the IC_50_ values of mutant cell lines by the average IC_50_ value of Ba/F3 cells expressing WT EGFR supplemented with 10 ng ml^−1^ EGF for each drug. Statistical differences between groups were determined by one-way ANOVA as described in the figure legends.

### In silico mutational mapping and docking experiments

X-ray structures of wild type EGFR in complex with AMP-PNP (2ITX) and osimertinib (4ZAU), and EGFR L858R mutant in complex with AMP-PNP (Protein Data Bank (PDB) ID: 2ITV) were retrieved from the Protein Data Bank. Molecular Operating Environment (2019.01; Chemical Computing Group CCCG) was used to generate mutant homology models, construct protein–ligand models and for visualization. Pymol was used for visualization of mutation location on WT EGFR (PDB ID: 2ITX) and structural alignment with EGFR D770insNPG (PDB ID: 4LRM) or EGFR G719S (PDB ID: 2ITN).

### Heat map generation

Heat maps and hierarchical clustering were generated by plotting the median log (Mut/WT) value for each cell line and each drug using R and the ComplexHeatmap package^40^ 2.6.2 (R Foundation for Statistical Computing). Hierarchical clustering was determined by Euclidean distance between Mut/WT ratios. For co-occurring mutations, mutation order was assigned arbitrarily, and for acquired mutations, mutations were assigned in the order mutations are observed clinically. Structure–function groups were assigned based on predicted impact of mutation on receptor conformation.

### Determination of EGFR groups and subgroups

Mutational mapping was used to separate EGFR mutations into distinct groups based on predicted drug sensitivity. Structural features of EGFR mutations with known drug sensitivity (that is, classical EGFR mutations^41,42^, T790M^43–45^ and exon 20 insertions^22,25^) were used as the basis for predicting the impact of mutations on drug sensitivity. Using mutational mapping there were four distinct groups: (1) no obvious effect on the drug binding pocket (similar to L858R); (2) a mutation in the hydrophobic core (similar to T790M); (3) a large inward shift of both the αC-helix and P-loop (similar to exon 20 insertions); and (4) a slight inward shift of the αC-helix and/or P-loop due to direct changes to the either the αC-helix and/or P-loop or indirectly through alterations of the ß-pleated sheets that are predicted to effect the position either the αC-helix and/or P-loop. Groups were validated by hierarchical clustering of in vitro sensitivity of Ba/F3 cells expressing the various EGFR mutations. Subgroups such as T790M-like-3S/T790M-like-3R and Ex20ins-NL/Ex20ins-FL were defined based on cell line sensitivity data.

### Statistical analyses of structure-function groups

Correlations for mutations were determined using Spearman’s rho by correlating the median log (Mut/WT) value for each mutation and drug versus the average of the median log (Mut/WT) value for the structure–function-based group or exon-based group for which the mutation belongs. For each correlation, the mutation tested was removed from the average structure function and exon-based groups. Average rho values were compared by two-sided Student’s *t*-test. To determine whether structure function groups or exon groups were better predictor of drug sensitivity, we performed recursive-partitioning analyses to construct a decision tree for each drug using structure function group and mutation data on exons 18, 19, 20, and 21 as predictors. The decision tree classified samples by posing a series of decision rules based on predictors. Each decision rule was constrained in an internal node, and every internal node points to yes-or-no questions that result in a ‘yes’ or ‘no’ branch. We applied the CART algorithm^20,21^ using the rpart R package. We calculated variable importance as the sum of the goodness of split measures for each split. These were scaled to sum to 100 for a tree. Median SAS version 9.4 and R version 3.5.6 were used to carry out the computations for all analyses. The structure function group variable was involved in the first and second splits in all of the 18 regression trees of drug sensitivity. The variable importance of this variable was in a range of 66–94%. Both the order of the split and variable importance indicate that the structure function group variable was more predictive than the exon-based variables in evaluation of drug sensitivity. Code for this analysis can be found at https://github.com/MD-Anderson-Bioinformatics/EGFR-Structure-Function-Nature-Manuscript.

### PDX generation and in vivo experiments

As part of the MD Anderson Cancer Center Lung Cancer Moon Shots program, PDXs harbouring EGFR G719A and EGFR L858R/E709K were generated and maintained in accordance with Good Animal Practices and with approval from MD Anderson Cancer Center Institutional Animal Care and Use Committee on protocol number PA140276 as previously described^46^. Surgical samples were rinsed with serum-free RPMI supplemented with 1% penicillin-streptomycin then implanted into the right flank of 5- to 6-week-old NSG female mice within 2 h of resection. Tumours were validated for EGFR mutations by DNA fingerprinting and quantitative PCR as described^46^. PDXs harbouring EGFR S768dupSVD were purchased from Jackson Laboratories (J100672). To propagate tumours, 5- to 6-week-old female NSG mice (NOD.Cg-Prkdcscid IL2rgtmWjl/Szj) were purchased from Jackson Laboratories (005557). Fragments of NSCLC tumours expressing EGFR S768dupSVD, G719A or L858R/E709K were implanted into 6- to 8-week-old female NSG mice. Once tumours reached 2,000 mm^3^, they were collected and re-implanted into the right flank of 6- to 8-week-old female NSG mice. Tumours were measured 3 times per week and were randomized into treatment groups when tumors reached a volume of 275–325 mm^3^ for the EGFR G719A and S768dupSVD models, and 150–175 mm^3^ for the L858R/E709K model. Treatment groups included vehicle control (0.5% methylcellulose, 0.05% Tween-80 in dH_2_O), 100 mg kg^−1^ erlotinib, 20 mg kg^−1^ afatinib, 2.5 mg kg^−1^ poziotinib, 5 mg kg^−1^ osimertinib, and 25 mg kg^−1^ osimertinib. During treatment, body weight and tumour volumes were measured three times per week, and mice received treatment five days per week (Monday to Friday). Dosing holidays were given if mouse body weight decreased by more than 10% or overall body weight dropped below 20 g. Maximum allowed tumour burden by approved IACUC protocol was a volume of 2,000 mm^3^. Mice were humanely euthanized if tumour sizes exceeded the maximum size.

### Case studies of patients treated with second-generation TKIs

Patients were consented under the GEMINI protocol (PA13-0589) which was approved in accordance with the MD Anderson Institutional Review Board, or protocol MCC 19161, which was formally reviewed and granted approval by Moffitt Cancer Center in accordance with the Declaration of Helsinki and the 21st Century Cures Act for retrospective analysis of patient outcomes and treatment course for case studies of patients presented. Both protocols include informed consent for publication of deidentified data.

### Retrospective analysis of ORR and duration of treatment with afatinib

Response to afatinib and duration of afatinib treatment was tabulated from 803 patients in the Uncommon EGFR Database (www.uncommonegfrmutations.com). Objective response rate was reported in 529 patients. Patients were stratified by either structure–function-based groups or exon-based groups and ORR was determined by counting the number of patients reported to have complete response or partial response. Fisher’s exact test was used to determined statistical differences between subgroups (structure based or exon-based). Duration of treatment was provided in the Uncommon EGFR Database for 746 patients. Patients were stratified by structure–function-based groups and exon-based groups and median DOT was calculated using the Kaplan–Meier method. Statistical differences in Kaplan–Meier plots, HR and *P* values were generated using GraphPad Prism software and the Mantel–Cox log-rank method. When mutations were not explicitly stated (that is, exon 19 mutation) those patients were excluded from the structure–function-based analysis but included in the exon-based analysis.

### Reporting summary

Further information on research design is available in the Nature Research Reporting Summary linked to this paper.

## Online content

Any methods, additional references, Nature Research reporting summaries, source data, extended data, supplementary information, acknowledgements, peer review information; details of author contributions and competing interests; and statements of data and code availability are available at 10.1038/s41586-021-03898-1.

## Supplementary information

Supplementary Tables This file contains Supplementary Tables 1–7.

Reporting Summary

## Data Availability

Source data for all figures can be found at https://github.com/MD-Anderson-Bioinformatics/EGFR-Structure-Function-Nature-Manuscript. Public datasets used in this study include non-overlapping studies including NSCLC in cBioportal (www.cbioportal.org) and the Uncommon EGFR Database (www.uncommonegfrmutations.com). Details of specific studies accessed can be found in the Reporting Summary. Data from Foundation Medicine and Guardant Health were provided under data use agreements; however, summarized data used in Fig. 1 and Extended Data Fig. 1 are provided at https://github.com/MD-Anderson-Bioinformatics/EGFR-Structure-Function-Nature-Manuscript. Source data are provided with this paper.

## Source data

Source Data Fig. 1
Source Data Fig. 2
Source Data Fig. 3
Source Data Fig. 4
Source Data Extended Data Fig. 1
Source Data Extended Data Fig. 3
Source Data Extended Data Fig. 4
Source Data Extended Data Fig. 5
Source Data Extended Data Fig. 6
Source Data Extended Data Fig. 7
Source Data Extended Data Fig. 8
Source Data Extended Data Fig. 10

## Code availability

All code used in this study can be found at https://github.com/MD-Anderson-Bioinformatics/EGFR-Structure-Function-Nature-Manuscript.
