# Supplementary material for: Structure-based classification predicts drug response in EGFR-mutant NSCLC
Source: Nature. 2021 Sep 15;597(7878):732–7. doi: 10.1038/s41586-021-03898-1 (PMC8481125; doi:10.1038/s41586-021-03898-1)
Supplement: Supplementary file 1 — This file contains Supplementary Tables 1–7. [file 41586_2021_3898_MOESM1_ESM.pdf]

nature portfolio

https://doi.org/10.1038/s41586-021-03898-1

Supplementary information

Structure-based classification predicts drug response in *EGFR*-mutant NSCLC

In the format provided by the authors and unedited

Supplemental Table 1. Summary of compounds

EGFR TKI	Class	Primary Target	Binding	Structure
Erlotinib	first-generation	EGFR	non-covalent	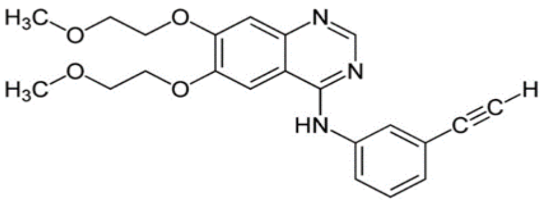
Gefitinib	first-generation	EGFR	non-covalent	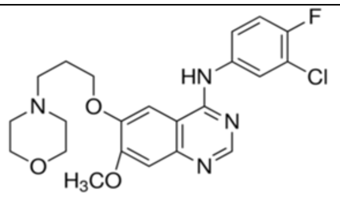
AZD3759	first-generation	EGFR	non-covalent	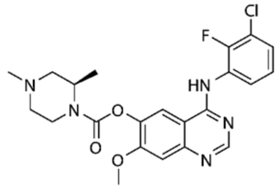
Sapatinib	first-generation	EGFR	non-covalent	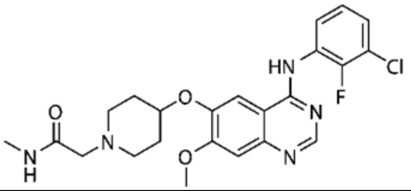
Afatinib	second-generation	EGFR	covalent	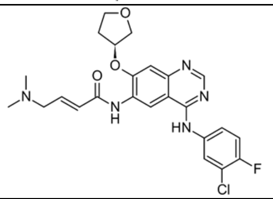
Dacomitinib	second-generation	EGFR	covalent	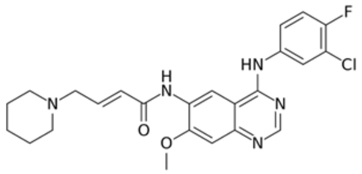
Neratinib	second-generation	EGFR	covalent	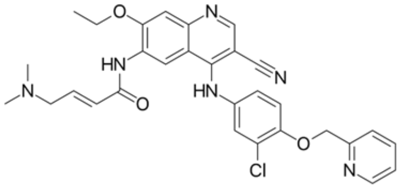
Poziotinib	second-generation	EGFR	covalent	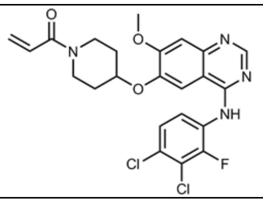

Tarlox-TKI	second-generation	EGFR	covalent	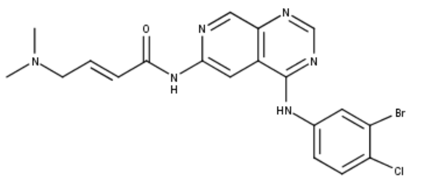
CLN-081	Ex20ins Active	EGFR	covalent	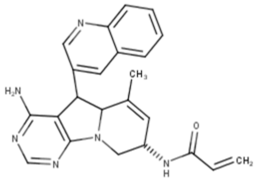
AZ5104	Ex20ins Active	EGFR	covalent	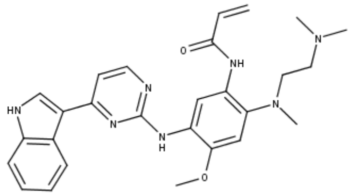
Mobocertinib	Ex20ins Active	EGFR	covalent	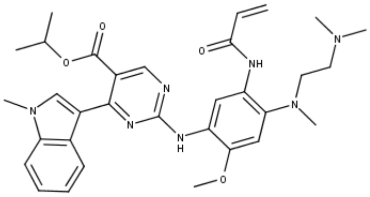
Osimertinib	third-generation	EGFR	covalent	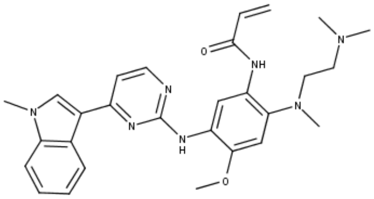
Nazartinib	third-generation	EGFR	covalent	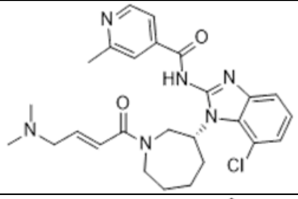
Olmutinib	third-generation	EGFR	covalent	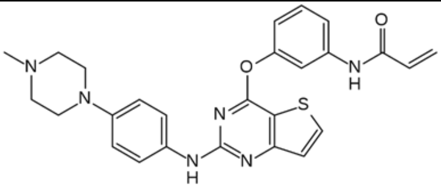
Rociletinib	third-generation	EGFR	covalent	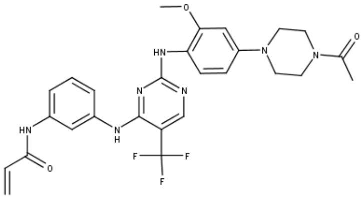
Naquotinib	third-generation	EGFR	covalent	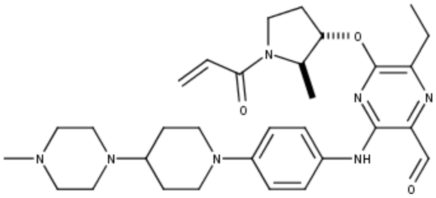

Lazertinib	third-generation	EGFR	covalent	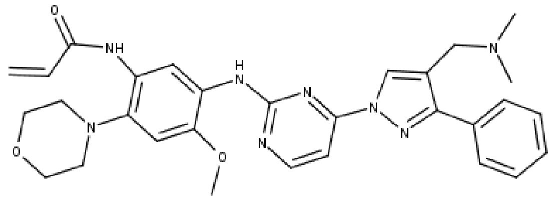
Ruboxistaurin	PKC	PKC	non-covalent	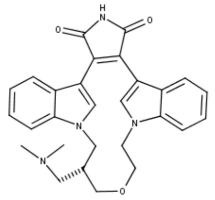
Sotrastaurin	PKC	PKC	non-covalent	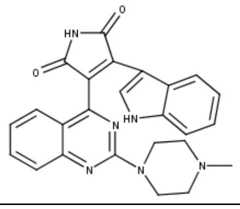
Midostaurin	PKC	PKC	non-covalent	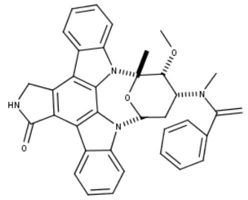
CUDC-101	EGFR/HDAC	EGFR/HDAC	non-covalent	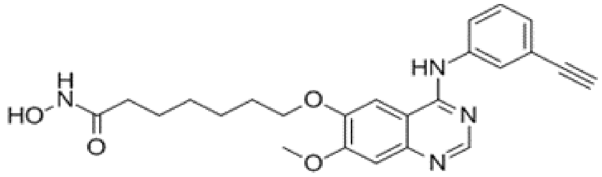
Brigatinib	ALK	ALK	non-covalent	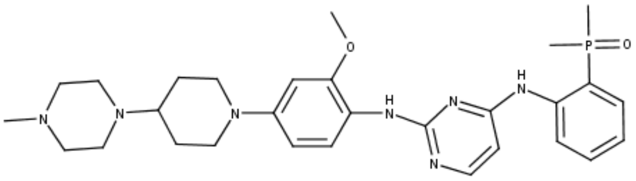
AZD3463	ALK	ALK	non-covalent	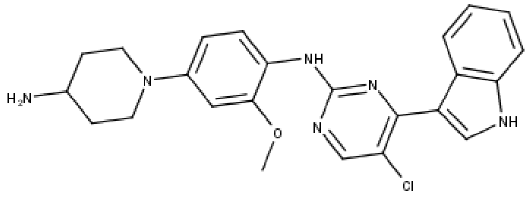

Supplemental Table 2. Summary of variable importance determined by CART including T790M-like mutations

Summary of variable importance		
**Drug**	**Structure function group**	**Exon-based group**
Erlotinib	78	22
Gefitinib	90	11
AZD3759	77	23
Sapatinib	77	23
Afatinib	75	25
Dacomitinib	75	25
Neratinib	66	34
Poziotinib	94	6
Tarlox-TKI	79	21
CLN-081	91	9
AZ5104	91	9
Mobocertinib	90	10
Osimertinib	87	14
Nazartinib	82	18
Olmutinib	89	11
Rociletinib	85	15
Naquotinib	78	22
Lazertinib	91	9

Supplemental Table 3. Summary of variable importance determined by CART without T790M-like mutations

**Summary of variable importance**		
**Drug**	**Structure function group**	**Exon-based group**
Erlotinib	85	3.75
Gefitinib	90	2.5
AZD3759	83	4.25
Sapatinib	77	5.5
Afatinib	80	5.25
Dacomitinib	83	4.25
Neratinib	51	12.25
Poziotinib	23	19.25
Tarlox-TKI	82	4.5
CLN-081	84	4.25
AZ5104	73	6.75
Mobocertinib	73	6.75
Osimertinib	88	5.5
Nazartinib	83	4.5
Olmutinib	85	3.75
Rociletinib	75	6.5
Naquotinib	82	4.5
Lazertinib	73	6.75

Supplemental Table 4: List of EGFR mutations and assigned subgroups used in manuscript

Classical-Like Mutations	PACC Mutations		Ex20 Loop Insertions		T790M-like Mutations	
			**Near-loop**	**Far-loop**	**T790M-like-3S**	**T790M-like-3R**
A702T	A750_I759del insPN	K757M L858R	A767_V769dup ASV	H773_V774 insNPH	Ex19del T790M	Ex19del T790M C797S
A763insFQEA	E709_T710del insD	K757R	A767_S768ins TLA	H773_V774 insAH	Ex19del T790M L718V	Ex19del T790M L792H
A763insLQEA	E709A	L718Q	S768_D770dup SVD	H773dupH	Ex19del T790M G724S	G724S T790M
D761N	E709A G719A	L718Q Ex19del	S768_D770dup SVD L858Q	V774_C775 insHV	G719A T790M	L718Q T790M
E709A L858R	E709A G719S	L718Q L858R	S768_D770dup SVD R958H	V774_C775 insPR	G719S T790M	L858R T790M C797S
E709K L858R	E709K	L718V	S768_D770dup SVD V769M		H773R T790M	L858R T790M L718Q
E746_A750del A647T	E709K G719S	L718V Ex19del	V769_D770ins ASV		I744_E749del insMKK	L858R T790M L718V
E746_A750del L41W	E736K	L718V L858R	V769_D770ins GSV		L747_K754 delinsATSPE	
E746_A750del R451H	E746_A750del A647T	L747_S752del A755D	V769_D770ins GVV		L858R T790M L792H	
Ex19del E746_A750del	E746_A750del R675W	L747P	V769_D770ins MASVD		L858R T790M V843I	
K754E	E746_T751del insV S768C	L747S	D770_N771ins NPG		L858R T790M	
L747_E749del A750P	Ex19del C797S	L747S L858R	D770_N771ins SVD		S768I T790M	
L747_T751del L861Q	Ex19del G796S	L747S V774M	D770del insGY		T790M	
L833F	Ex19del L792H	L858R C797S	D770_N771 insG			
L833V	Ex19del T854I	L858R L792H	D770_N771 insY H773Y			
L858R	G719A	L858R T854S	N771dupN			
L858R A289V	G719A D761Y	N771G	N771dupN G724S			
L858R E709V	G719A L861Q	R776C	N771_P772ins HH			
L858R L833F	G719A R776C	R776H	N771_P772ins SVDNR			
L858R P100T	G719A S768I	E709_T710del insD S22R	P772_H773ins DNP			
L858R P848L	G719A S768I	S752_I759del V769M				
L858R R108K	G719C S768I	S768I				
L858R R324H	G719S	S768I L858R				
L858R R324L	G719S L861Q	S768I L861Q				
L858R S784F	G719S S768I	S768I V769L				
L858R S784Y	G724S	S768I V774M				
L858R T725M	G724S Ex19del	T751_I759 delinsN				
L858R V834L	G724S L858R	V769L				
L861Q	G779F	V769M				
L861R	I740dupIPVAK	V774M				
S720P						
S784F						
S811F						
T725M						

Supplemental Table 5: Patient Characteristics

Mutation Type	Exon	EGFR Mutation	Age (years)	Sex	Tobacco Use (Y/N)	Pack Years	Histo Pathology	Previous Chemo (Y/N)	TKI	Stage at diagnosis
Classical Like	19	E746_A750 A750E	73	M	Y	Unknown	Adenocarcinoma	N	Erlotinib	IV
Classical Like	21	L747_T751del L861Q	59	F	Y	5	Adenocarcinoma	N	Afatinib	IV
Classical Like	21	L861Q	70	F	N	N/A	Adenocarcinoma	N	Afatinib	II
Classical Like	21	L858R E709K	59	F	Y	10	Adenocarcinoma	N	Afatinib	IV
Classical Like	21	L858R P848L	74	M	N	N/A	Adenocarcinoma	N	Osimertinib	IV
Classical Like	18	L858R T725M	52	F	N	N/A	Adenocarcinoma	N	Osimertinib	IV
Classical Like	21	L861Q	77	M	N	N/A	Adenocarcinoma	N	Osimertinib	IV
Classical Like	21	L861Q	78	M	N	N/A	Adenocarcinoma	Y	Erlotinib	IV
Classical Like	18	T725M	75	F	N	N/a	Adenocarcinoma	Y	Gefitinib	IV
Classical Like	18	A702T	62	M	Y	47	Adenocarcinoma	Y	Afatinib	IV
Classical Like	21	S811F	84	F	Y	37	Adenocarcinoma	Y	erlotinib	IV
Classical Like	19	L747_E749del A750P	81	F	Y	8	Adenocarcinoma	N	Erlotinib	I
Classical Like	21	L861Q	70	M	Y	24	Adenocarcinoma	N	Erlotinib	IV
Classical Like	21	L861Q	73	F	N	N/A	Adenocarcinoma	N	Erlotinib	I
Classical Like	21	L858R L833F	82	M	Y	51	Adenocarcinoma	Y	Erlotinib	IV
Classical Like	18	L858R E709V	60	M	N	N/A	Adenocarcinoma	Y	Rociletinib	IV
Classical Like	19	E746_A750del A647T	56	M	N	N/A	Adenocarcinoma	Y	Erlotinib	IV
Classical Like	21	E709K L858R	70	M	Y	5	Adenocarcinoma	Y	Erlotinib	IV
Classical Like	21	L861Q	82	F	Y	Unknown	Adenocarcinoma	N	Erlotinib	IV
Classical Like	18	E709K L858R	45	M	N	N/A	Adenocarcinoma	N	Rociletinib	IV
Classical Like	21	L861Q	60	M	Y	90	Adenocarcinoma	N	Gefitinib	IV
Classical Like	21	E709K/L858R	83	F	N	N/A	Adenocarcinoma	N	Afatinib	IV
Classical Like	19	L747_E749del A750P	45	M	Y	5	Adenocarcinoma	Y	Osimertinib	III
Classical Like	21	L861Q	77	F	Y	10	Adenocarcinoma	N	Erlotinib	IV
Classical Like	19	D761Y	74	M	Y	35	Squamous cell carcinoma	Y	Afatinib	IV
Classical Like	21	L858R S784F	71	F	N	N/A	Adenocarcinoma	N	Osimertinib	IV
Classical Like	21	L858R V834L	75	F	Y	20	Adenocarcinoma	Y	Osimertinib	III
Classical Like	21	L858R L833V	60	F	N	N/A	Adenocarcinoma	Y	Osimertinib	IV
Classical Like	21	L861Q	71	F	N	N/A	Adenocarcinoma	N	Osimertinib	IV
Classical Like	18	E709A L858R	69	M	N	N/A	Adenocarcinoma	Y	Osimertinib	IV
Classical Like	21	L861Q	79	M	N	N/A	Adenocarcinoma	N	Osimertinib	IV

Classical Like	21	L858R S784Y	53	M	Y	15	Adenocarcinoma	N	Osimertinib	IV
Classical Like	6	L858R R324L	71	F	Y	32	Adenocarcinoma	Y	Erlotinib	IV
Classical Like	5	L858R A289V	57	M	N	N/A	Adenocarcinoma	N	Erlotinib	IV
Classical Like	6	L858R R324H	70	F	N	N/A	Adenocarcinoma	Y	Erlotinib	IV
Classical Like	1	L858R R108K	73	F	N	N/A	Adenocarcinoma	N	Erlotinib	IV
Classical Like	1	E746_A750del L41W	59	F	N	N/A	Adenocarcinoma	N	Gefitinib	IV
Classical Like	1	L858R P100T	65	M	Y	43	Adenocarcinoma	N	Gefitinib	IV
Classical Like	9	E746_A750del R451H	57	F	N	N/A	Adenocarcinoma	N	Erlotinib	IV
Ex20LoopIns	20	S768_D770dupSVD R958H	69	F	Unknown	1	Adenocarcinoma	Y	Poziotinib	I
Ex20LoopIns	20	S768_D770dupSVD V769M	63	M	N	N/A	Adenocarcinoma	Y	Poziotinib	IV
Ex20LoopIns	20	S768_D770dupSVD L858Q	50	M	N	N/A	Adenocarcinoma	Y	Poziotinib	IV
Ex20LoopIns	20	Exon 20 insertion	63	F	N	N/A	Adenocarcinoma	N	Osimertinib	IV
Ex20LoopIns	20	H773_V774insH	60	M	N	NA	Adenocarcinoma	Y	Erlotinib	IV
Ex20LoopIns	20	A767_v769dupASV	61	F	N	NA	Adenocarcinoma	Y	Erlotinib	IV
Ex20LoopIns	20	D770_N771insG	49	F	N	NA	Adenocarcinoma	Y	Afatinib	IV
Ex20LoopIns	20	N771delinsGY	79	M	N	NA	Adenocarcinoma	Y	Erlotinib	IV
Ex20LoopIns	20	S768_D770dupSVD	47	F	N	NA	Adenocarcinoma	N	Erlotinib	IV
Ex20LoopIns	20	A767_V769dupASV	69	M	Y	35	Adenocarcinoma	Y	Afatinib	IV
Ex20LoopIns	20	S768_D770dupSVD	65	M	Y	0.75	Adenocarcinoma	N	Erlotinib	IV
Ex20LoopIns	20	S768_V769delinsIL	57	F	Y	<5	Adenocarcinoma	Y	Afatinib	IV
Ex20LoopIns	20	S768_D770dupSVD	56	F	N	NA	Adenocarcinoma	N	Erlotinib	IV
PACC	18	G719S	65	F	Y	60	Adenocarcinoma	N	Osimertinib	III
PACC	18	G719A S768I	47	M	N	N/A	Adenocarcinoma	N	Erlotinib	IV
PACC	18	G719S S768I	43	F	Y	2	Adenocarcinoma	N	Afatinib	I
PACC	19	L858R K757M	60	F	Y	5	Adenocarcinoma	N	Erlotinib	I
PACC	18	G719S L861Q	55	F	Y	10	Adenocarcinoma	Y	Gefitinib	III
PACC	18	G719A	85	M	Y	50	Adenocarcinoma	N	Afatinib	IV
PACC	18	G719A	66	F	Y	12	Adenocarcinoma	N	Osimertinib	IV
PACC	19	L747_S752del A755D	62	F	Y	25	Adenocarcinoma	N	Osimertinib	IV
PACC	20	V769M	89	M	Y	45	Adenosquamous	N	Osimertinib	IV
PACC	18	E709_T719del insD	78	F	N	N/A	Adenocarcinoma	N	Afatinib	IV
PACC	18	G719A	62	M	Y	10	Adenocarcinoma	Y	Erlotinib	IV
PACC	21	L858R T854S	71	F	Y	Unknown	Adenosquamous	N	Afatinib	IV

PACC	18	G719C S768I	70	F	Y	15	Adenocarcinoma	N	Erlotinib	IV
PACC	20	L858R S768I	82	M	Y	45	Adenocarcinoma	Y	Afatinib	III
PACC	20	L858R S768I	85	F	N	N/A	Adenocarcinoma	N	Osimertinib	IV
PACC	18	G719A	78	M	Y	24	Adenocarcinoma	N	Afatinib	IV
PACC	18	G719S S768I	74	M	N	N/A	Adenocarcinoma	N	Erlotinib	IV
PACC	19	L858R L747S	69	M	N	N/A	Adenocarcinoma	N	Erlotinib	IV
PACC	20	E746_T751delinsV S768C	74	F	N	N/A	Adenocarcinoma	N	Erlotinib	IV
PACC	18	E709A G719A	74	M	Y	chewing tobacco	Adenocarcinoma	Y	Afatinib	IV
PACC	20	S752_I759del V769M	64	M	N	N/A	Adenocarcinoma	Y	Erlotinib	IV
PACC	18	G719S S768I	75	F	N	N/A	Adenocarcinoma	N	Erlotinib	IV
PACC	18	G719A S768I	60	M	Y	90	Adenocarcinoma	Y	Erlotinib	IV
PACC	18	G719A S768I	66	M	N	N/A	Adenocarcinoma	Y	Poziotinib	IV
PACC	18	G719A S768I	66	M	N	N/A	Adenocarcinoma	Y	Avitinib	IV
PACC	18	E709_T710delinsD S22R	80	M	Y	22	Adenocarcinoma	Y	Afatinib	I
PACC	19	E746_A750del R675W	66	M	N	N/A	Adenocarcinoma	Y	Erlotinib	IV
PACC	18	G719A	69	F	N	N/A	Adenocarcinoma	N	Erlotinib	IV
PACC	19	E736K	59	F	Y	14.5	Adenocarcinoma	Y	Gefitinib	III
PACC	18	G719A S768I	75	F	Y	17	Adenocarcinoma	Y	Afatinib	III
PACC	18	E709_T710delinsD	66	M	N	N/A	Adenocarcinoma	Y	Afatinib	III
PACC	18	G719A	66	M	Y	25	Adenocarcinoma	Y	Afatinib	III
PACC	18	G719A	68	F	Y	25	Adenocarcinoma	N	Afatinib	III
PACC	19	T751_I759delinsN	59	F	N	N/A	Adenocarcinoma	Y	Gefitinib	IV
PACC	20	L861Q S768I	56	F	Y	38	Adenocarcinoma	Y	Afatinib	IV
PACC	19	L747S V774M	49	F	Y	15	Adenocarcinoma	N	Afatinib	IV
PACC	20	G719A R776C	52	F	N	N/A	Adenocarcinoma	N	Erlotinib	IV
PACC	18	E709K G719S	62	F	N	N/A	Adenocarcinoma	N	Afatinib	IV
PACC	20	N771G	60	F	N	N/A	Adenocarcinoma	N	Poziotinib	IV
PACC	18	G719A	87	F	Y	17.5	Adenocarcinoma	Y	Osimertinib	IV
PACC	18	E709K G719S	84	F	N	N/A	Adenocarcinoma	Y	Afatinib	IV
PACC	18	G719A	67	M	N	N/A	Adenosquamous	N	Afatinib	III
PACC	20	G779F	71	F	Y	24	Adenocarcinoma	Y	Afatinib	IV
PACC	19	T751_I759delinsN	74	F	Y	1	Adenocarcinoma	Y	Osimertinib	IV
PACC	19	L747P	63	F	N	N/A	Adenocarcinoma	N	Osimertinib	I
PACC	18	G719S S768I	62	M	N	N/A	Adenocarcinoma	Y	Afatinib	IV

PACC	19	A750_I759delinsPN	51	F	Y	13	Squamous cell carcinoma	Y	Osimertinib	IV
PACC	18	G719A D761Y	67	F	Y	32	Adenocarcinoma	Y	Osimertinib	II
PACC	20	S768I	64	F	Y	15	Adenocarcinoma	N	Afatinib	IV
PACC	18	E709A G719S	45	F	N	N/A	Adenocarcinoma	N	Afatinib	IV
PACC	18	G719S L861Q	66	M	N	N/A	Adenocarcinoma	N	Afatinib	IV
PACC	18	G719A	68	F	N	N/A	Adenocarcinoma	Y	erlotinib	IV
PACC	18	G719A	71	F	N	N/A	Large Neuroendocrine	N	Afatinib	IV
T790M-like-3S	18	G719S T790M	62	F	N	N/A	Adenocarcinoma	N	Osimertinib	IV
T790M-like-3S	20	H773R T790M	50	F	N	N/A	Adenocarcinoma	Y	Osimertinib	IV
T790M-like-3S	18	G719A T790M	60	F	N	N/A	Adenocarcinoma	N	Osimertinib	IV
T790M-like-3S	19	I744_E749delinsMKK	60	F	N	N/A	Adenocarcinoma	N	Osimertinib	IV

Supplemental Table 6: Patient Characteristics stratified by EGFR mutation type

Clinical factors	Classical-Like	PACC	Ex20LoopIns	T790M-like	*P* [a]
Total n (%)	39 (36)	53 (49)	13 (12)	4 (3)	
Age, y, median (range)	70 (45-84)	66 (43-89)	61(47-79)	60 (50-62)	> 0.9999
< 65	14 (36)	20 (38)	9 (69)	4	
>=65	25 (64)	33 (62)	4 (31)	0	
Sex n (%)					0.3934
Male	19 (49)	20 (38)	6 (46)	0	
Female	20 (51)	33 (62)	7 (54)	4	
Smoking status n (%)					0.6733
Smokers	18 (46)	28 (53)	3 (23)	0	
Nonsmokers	21 (54)	25 (47)	9 (69)	4	
Unknown	0	0	1 (8)	0	
Stage n (%)					0.1268
I-III	5 (13)	14 (26)	1 (8)	0	
IV	34 (87)	39 (74)	12 (92)	4	
Histology n (%) [b]					> 0.9999
ADC	38 (97)	48 (90)	13	4	
SCC	1 (3)	1 (2)	0	0	
ASC	0	3 (6)	0	0	
LCNEC	0	1 (2)	0	0	

The associations of EGFR mutation type with clinical variables were evaluated by Fisher's exact test. Statistical significance was considered for $p < 0.05$ and all tests were two-tailed.

[a]Comparison between Classical Like and PACC

[b]Comparison between ADC and SCC

Supplemental Table 7: EGFR mutant vectors used to generate cell lines

EGFR Mutation	Starting Vector	Genomic change	Manufacturer	Purchased / Created
Ex19del (E746_A750del)	N/A	N/A	Addgene #32062	Purchased
WT	N/A	N/A	Addgene #11011	Purchased
L858R	N/A	N/A	Addgene #11012	Purchased
G719S	N/A	N/A	Addgene #11013	Purchased
D770insNPG	N/A	N/A	Addgene #11016	Purchased
A767insASV	N/A	N/A	Addgene #32066	Purchased
L861Q	N/A	N/A	Addgene #32068	Purchased
T790M	N/A	N/A	Addgene #32070	Purchased
Ex19del T790M	N/A	N/A	Addgene #32072	Purchased
L858R/T790M	N/A	N/A	Addgene #32073	Purchased
A763insFQEA	EGFR WT	c.2290_2291insTCCAGGAAGCCT	Bioinnovatise	Created
A767insTLA	EGFR WT	c.2302_2303insCGCTGGCCA	Bioinnovatise	Created
D770del insGY	EGFR WT	c.2308_2309insGTT	Bioinnovatise	Created
D770insG	EGFR WT	c.2310_2311insGGT	Bioinnovatise	Created
D770insY H773Y	EGFR WT	c.2310_2311insTAC and c.2317C>T	Bioinnovatise	Created
Ex19del C797S	EGFR Ex19del	c.2386, T>A	Bioinnovatise	Created
Ex19del L718Q	EGFR Ex19del	c.2153T>A	Bioinnovatise	Created
Ex19del L718V	EGFR Ex19del	c.2152C>G	Bioinnovatise	Created
Ex19del L792H	EGFR Ex19del	c.2375T>A	Bioinnovatise	Created
Ex19del T790M C797S	EGFR Ex19del T790M	c.2386, T>A	Bioinnovatise	Created
Ex19del T790M G724S	EGFR Ex19del T790M	c.2313_2314insAAC	Bioinnovatise	Created
Ex19del T790M L718V	EGFR Ex19del T790M	c.2152C>G	Bioinnovatise	Created
Ex19del T790M L792H	EGFR Ex19del T790M	c.2375T>A	Bioinnovatise	Created
G719A	EGFR WT	c.2156G>C	Bioinnovatise	Created
G719A L861Q	EGFR G719A	c.2582T>A	Bioinnovatise	Created
G719A T790M	EGFR T790M	c.2156G>C	Bioinnovatise	Created
H773insAH	EGFR WT	c.2320_2321insCCCACG	Bioinnovatise	Created
H773insH	EGFR WT	c.2319_2320insCAC	Bioinnovatise	Created
H773insNPH	EGFR WT	c.2319_2320insAACCCCCAC	Bioinnovatise	Created
I740dupIPVAK	EGFR WT	c.2214_2231dup	Bioinnovatise	Created
L858R C797S	EGFR L858R	c.2386, T>A	Bioinnovatise	Created
L858R L792H	EGFR L858R	c.2375T>A	Bioinnovatise	Created
L858R T790M C797S	EGFR L858R T790M	c.2386, T>A	Bioinnovatise	Created
L858R T790M L792H	EGFR L858R T790M	c.2375T>A	Bioinnovatise	Created
L858R V834L	EGFR L858R	c.2500G>T	Bioinnovatise	Created
N771insHH	EGFR WT	c.2314_2315insACCACC	Bioinnovatise	Created
N771insSVDNR	EGFR WT	c.2312_2315ACCC>gcgtggacaaccg	Bioinnovatise	Created
P772insDNP	EGFR WT	c.2316_2317ins gacaacccc	Bioinnovatise	Created

S768dupSVD	EGFR WT	c.2303_2304dupAGCG TGGAC	Bioinnovatise	Created
S768I	EGFR WT	c.2303G>T	Bioinnovatise	Created
S768I T790M	EGFR S768I	c.2369C>T	Bioinnovatise	Created
V769insASV	EGFR WT	c.2308_2309insCCAG CGTGG	Bioinnovatise	Created
V769insGSV	EGFR WT	c.2308_2309insGCAG CGTGG	Bioinnovatise	Created
V769insGVV	EGFR WT	c.2308_2309insGGGT CGTGG	Bioinnovatise	Created
V769insMASVD	EGFR WT	c.2307_2308ins atggccagcgtggac	Bioinnovatise	Created
V769L	EGFR WT	c.2305G>T	Bioinnovatise	Created
V774insHV	EGFR WT	c.2321_2322insCCAC GT	Bioinnovatise	Created
V774insPR	EGFR WT	c.2322_2323insCCAC GT	Bioinnovatise	Created
A763insLQEA	EGFR WT	c.2289_2290insCTCC AGGAAGCC	GeneScript	Created
D761N	EGFR WT	c.2281G>A	GeneScript	Created
E709_T710del insD	EGFR WT	c.2127_2129del	GeneScript	Created
E709A	EGFR WT	c.2126A>C	GeneScript	Created
E709A G719S	EGFR G719S	c.2126A>C	GeneScript	Created
E709K	EGFR WT	c.2125G>A	GeneScript	Created
E709K G719S	EGFR G719S	c.2125G>A	GeneScript	Created
E709K L858R	EGFR L858R	c.2125G>A	GeneScript	Created
Ex19del G724S	EGFR Ex19del	c.2313_2314insAAC	GeneScript	Created
Ex19del G796S	EGFR Ex19del	c.2386G>A	GeneScript	Created
Ex19del T854I	EGFR Ex19del	c.2561C>T	GeneScript	Created
G719A/R776C	EGFR G719A	c.2326C>T	GeneScript	Created
G724S	EGFR WT	c.2170G>A	GeneScript	Created
G724S T790M	EGFR T790M	c.2313_2314insAAC	GeneScript	Created
K754E	EGFR WT	c.2260A>G	GeneScript	Created
K757R	EGFR WT	c.2270A>G	GeneScript	Created
L718Q	EGFR WT	c.2153T>A	GeneScript	Created
L718Q T790M	EGFR T790M	c.2153T>A	GeneScript	Created
L718V	EGFR WT	c.2152C>G	GeneScript	Created
L747_K754del insATSPE	EGFR WT	c.2239_2260delinsGC AACATCTCCGG	GeneScript	Created
L747P	EGFR WT	c.2239_2240TT>CC	GeneScript	Created
L747S	EGFR WT	c.2240T>C	GeneScript	Created
L833F	EGFR WT	c.2499G>T	GeneScript	Created
L833V	EGFR WT	c.2497T>G	GeneScript	Created
L858R G724S	EGFR L858R	c.2313_2314insAAC	GeneScript	Created
L858R G796S	EGFR L858R	c.2386G>A	GeneScript	Created
L858R L718Q	EGFR L858R	c.2153T>A	GeneScript	Created
L858R L718V	EGFR L858R	c.2152C>G	GeneScript	Created
L858R S784F	EGFR L858R	c.2351C>T	GeneScript	Created
L858R T790M L718Q	EGFR L858R T790M	c.2153T>A	GeneScript	Created
L858R T790M L718V	EGFR L858R T790M	c.2152C>G	GeneScript	Created

L858R T790M V843I	EGFR L858R T790M	c.2527G>A	GeneScript	Created
L861R	EGFR WT	c.2582T>G	GeneScript	Created
N771dupN G724S	EGFR G724S	c.2313_2314insAAC	GeneScript	Created
R776C	EGFR WT	c.2326C>T	GeneScript	Created
R776H	EGFR WT	c.2327G>A	GeneScript	Created
S720P	EGFR WT	c.2158T>C	GeneScript	Created
S768dupSVD V769M	EGFR S768dupSVD	c.2305G>A	GeneScript	Created
S768I V769L	EGFR S768I	c.2305G>T	GeneScript	Created
S768I V774M	EGFR S768I	c.2320G>A	GeneScript	Created
S784F	EGFR WT	c.2351C>T	GeneScript	Created
S811F	EGFR WT	c.2432C>T	GeneScript	Created
T725M	EGFR WT	c.2174C>T	GeneScript	Created
V774M	EGFR WT	c.2320G>A	GeneScript	Created
