# Supplementary material for: Structure-based classification predicts drug response in EGFR-mutant NSCLC
Source: Nature. 2021 Sep 15;597(7878):732–7. doi: 10.1038/s41586-021-03898-1 (PMC8481125; doi:10.1038/s41586-021-03898-1)
Supplement: Supplementary file 2 — Reporting Summary [file 41586_2021_3898_MOESM2_ESM.pdf]

nature research

Corresponding author(s): John V. Heymach, MD, Ph.D.

Last updated by author(s): Jul 20, 2021

Reporting Summary

Nature Research wishes to improve the reproducibility of the work that we publish. This form provides structure for consistency and transparency in reporting. For further information on Nature Research policies, see our Editorial Policies and the Editorial Policy Checklist.

Statistics

For all statistical analyses, confirm that the following items are present in the figure legend, table legend, main text, or Methods section.

n/a	Confirmed	
☐	☒	The exact sample size (*n*) for each experimental group/condition, given as a discrete number and unit of measurement
☐	☒	A statement on whether measurements were taken from distinct samples or whether the same sample was measured repeatedly
☐	☒	The statistical test(s) used AND whether they are one- or two-sided<br>*Only common tests should be described solely by name; describe more complex techniques in the Methods section.*
☒	☐	A description of all covariates tested
☐	☒	A description of any assumptions or corrections, such as tests of normality and adjustment for multiple comparisons
☐	☒	A full description of the statistical parameters including central tendency (e.g. means) or other basic estimates (e.g. regression coefficient) AND variation (e.g. standard deviation) or associated estimates of uncertainty (e.g. confidence intervals)
☐	☒	For null hypothesis testing, the test statistic (e.g. *F*, *t*, *r*) with confidence intervals, effect sizes, degrees of freedom and *P* value noted<br>*Give P values as exact values whenever suitable.*
☒	☐	For Bayesian analysis, information on the choice of priors and Markov chain Monte Carlo settings
☐	☒	For hierarchical and complex designs, identification of the appropriate level for tests and full reporting of outcomes
☐	☒	Estimates of effect sizes (e.g. Cohen's *d*, Pearson's *r*), indicating how they were calculated

Our web collection on statistics for biologists contains articles on many of the points above.

Software and code

Policy information about availability of computer code

Data collection	MOE (Molecular Operating Environment, 2019.01; Chemical Computing Group CCCG) was used to generate mutant homology models, construct protein-ligand models, and for visualization. Pymol version 4.6.0 - Build 26.20.100.7926 software was used for visualization of mutation location on WT (2ITX) EGFR, and structural alignment with EGFR D770insNPG (4LRM) or EGFR G719S (2ITN). Crystal structures were retrieved from PDB (www.rcsb.org).
Data analysis	Heat maps and hierarchical clustering were generated by plotting the median log (Mut/WT) value for each cell line and each drug using R and the ComplexHeatmap package 2.6.2 (R Foundation for Statistical Computing, Vienna, Austria. Complex Heatmap). Hierarchical clustering was determined by Euclidean distance between Mut/WT ratios. To determine if structure function groups or exon groups were better predictor of drug sensitivity, we performed recursive-partitioning analyses to construct a decision tree for each drug Using structure function group, mutation data on exons 18, 19, 20, and 21 as predictors. Decision tree classified samples by posing a series of decision rules based on predictors. Each decision rule was constrained in an internal node, and every internal node points to yes-or-no questions that result in a 'yes' or 'no' branch. We applied the classification and regression trees (CART) algorithm using "rpart" R package 4.1-15. We calculated variable importance as the sum of the goodness of split measures for each split. These are scaled to sum to 100 for a tree. Median SAS version 9.4 and R version 6.5.6 are used to carry out the computations for all analyses. Where indicated, analysis of heat maps was completed using ComplexHeatmap software package in R, and CART algorithm was applied using rpart in R package. Median SAS version 9.4 and R version 6.5.6 are used to carry out the computations of the CART algorithm. All other statistical analyses were completed using GraphPad Prism version 8.0. Code used for data analysis can be found at https://github.com/MD-Anderson-Bioinformatics/EGFR-Structure-Function-Nature-Manuscript.

For manuscripts utilizing custom algorithms or software that are central to the research but not yet described in published literature, software must be made available to editors and reviewers. We strongly encourage code deposition in a community repository (e.g. GitHub). See the Nature Research guidelines for submitting code & software for further information.

Data

Policy information about availability of data

All manuscripts must include a data availability statement. This statement should provide the following information, where applicable:

- Accession codes, unique identifiers, or web links for publicly available datasets
- A list of figures that have associated raw data
- A description of any restrictions on data availability

Source data for all figures is available at (https://github.com/MD-Anderson-Bioinformatics/EGFR-Structure-Function-Nature-Manuscript). Public datasets used in this study include non-overlapping studies including non-small cell lung cancer in cBioportal (www.cbioportal.org) including Broad, Cell 2012; MSKCC, 2020; MSKCC, Science 2015; NPJ Precision Oncology, MSK 2021; OncoSG, Nat Genet 2020; TCGA, Firehose Legacy; TSP, Nature 2008; MSKCC, Cancer Discov 2017; MSK, Cancer Cell 2018; MSKCC, J Clin Oncol 2018; TRACERx, NEJM & Nature 2017; University of Turin, Lung Cancer 2017; MSK, Science 2015; and TCGA, Nat Genet 2016Broad, Cell 2012; MSKCC, 2020; MSKCC, Science 2015; NPJ Precision Oncology, MSK 2021; OncoSG, Nat Genet 2020; TCGA, Firehose Legacy; TSP, Nature 2008; MSKCC, Cancer Discov 2017; MSK, Cancer Cell 2018; MSKCC, J Clin Oncol 2018; TRACERx, NEJM & Nature 2017; University of Turin, Lung Cancer 2017; MSK, Science 2015; and TCGA, Nat Genet 2016. Additional data was accessed from the uncommon EGFR database (www.uncommonegfrmutations.com). Data from Foundation Medicine and Guardant Health were provided under data use agreements; however, summarized data used in Figure 1 and Extended Figure 1 are provided at GitHub (https://github.com/MD-Anderson-Bioinformatics/EGFR-Structure-Function-Nature-Manuscript).

Field-specific reporting

Please select the one below that is the best fit for your research. If you are not sure, read the appropriate sections before making your selection.

☒ Life sciences ☐ Behavioural & social sciences ☐ Ecological, evolutionary & environmental sciences

For a reference copy of the document with all sections, see nature.com/documents/nr-reporting-summary-flat.pdf

Life sciences study design

All studies must disclose on these points even when the disclosure is negative.

Sample size	Sample size in vitro was determined by expected effect size, and the number of previously reported reoccurring atypical mutations. No statistical methods were used to predetermine sample size in vitro. In vivo, preliminary studies and previous studies of variability in tumor responses was used to determined N=5-6 was typically sufficient for determining significant differences (Robichaux et al 2018 Nat Med and Robichaux et al 2019 Cancer Cell).
Data exclusions	For the in vivo studies throughout the manuscript, mice that were humanly euthanized due to weight loss >20% were excluded from the analysis. This was a predetermined exclusion criteria. For the patient analysis in figure 4, patients receiving additional treatment during the time of TKI treatment (i.e. radiation, anti-VEGF, surgery) or lacking molecular data (i.e. EGFR mutation unknown) were excluded from the analysis. This was a predetermined exclusion criteria for the retrospective analysis.
Replication	In vitro studies were completed in biological triplicate without greater than expected variability, and no experiments were excluded. In vivo studies were completed with indicated biological replicates as noted in the figure legends and methods.
Randomization	For in vitro analysis of drug sensitivity, groups were assigned by mutation location (exon) or structure/function based groups. Mice were continuously randomized into treatment groups when tumors reached the indicated tumor sizes in the Methods section. For retrospective analyses of patient outcomes (TTF, DOT) patients were stratified by mutation location (exon) or structure/function based groups. For patients or cell lines with more than one mutation, the order of mutations were assigned randomly or by natural history (1. primary, 2. acquired) of the patient.
Blinding	Investigators were not blinded for in vitro or in vivo studies due to requirements of cage labeling and proper drug administration.

Reporting for specific materials, systems and methods

We require information from authors about some types of materials, experimental systems and methods used in many studies. Here, indicate whether each material, system or method listed is relevant to your study. If you are not sure if a list item applies to your research, read the appropriate section before selecting a response.

Materials & experimental systems

n/a	Involved in the study	
☒	☐	Antibodies
☐	☒	Eukaryotic cell lines
☒	☐	Palaeontology and archaeology
☐	☒	Animals and other organisms
☐	☒	Human research participants
☒	☐	Clinical data
☒	☐	Dual use research of concern

Methods

n/a	Involved in the study	
☒	☐	ChIP-seq
☒	☐	Flow cytometry
☒	☐	MRI-based neuroimaging

Eukaryotic cell lines

Policy information about cell lines

Cell line source(s)	Ba/F3 cells were a gift from Dr. Gordon Mills (The University of Texas, MD Anderson Cancer Center) from the MD Anderson Characterized Cell Line Core Facility .<br>Phoenix 293T-ampho cells were purchased from ATCC (CRL-3213).
Authentication	Cell line identity was confirmed by DNA fingerprinting via short tandem repeats using the PowerPlex 1.2 kit (Promega). Fingerprinting results were compared with reference fingerprints maintained by the primary source of the cell line.
Mycoplasma contamination	Regular mycoplasma testing is completed in our lab and cells were found to be free from mycoplasma.
Commonly misidentified lines (See ICLAC register)	No commonly misidentified cell lines were used in this study.

Animals and other organisms

Policy information about studies involving animals; ARRIVE guidelines recommended for reporting animal research

Laboratory animals	For PDX models, female NSG mice were engrafted with tumor fragments at 6-8 weeks of age. Five to eight mice were implanted per group. Mice with tumors not meeting the indicated tumor sizes were not randomized. Mice were housed in a modified barrier, specific pathogen free facility including automatic 12 hour light/dark cycles. The facility is kept at an ambient temperature of 72 degrees Fahrenheit and 45% humidity. Food and water are provided ad libitum.
Wild animals	This study did not include wild animals.
Field-collected samples	This study did not include field-collected samples.
Ethics oversight	Patient derived xenografts were generated and maintained in accordance with Good Animal Practices and with approval from MD Anderson Cancer Center Institutional Animal Care and Use Committee (Houston, TX) on protocol number PA140276.

Note that full information on the approval of the study protocol must also be provided in the manuscript.

Human research participants

Policy information about studies involving human research participants

Population characteristics	There were no co-variate analyses completed based on patient characteristics in this study.
Recruitment	Patients were consented prospectively through the MD Anderson GEMINI protocol. Recruitment for the GEMINI protocol is preformed at routine visits. Patients within the institution who are scheduled for appointments in the Thoracic Center will be identified by either an attending physician or by a study member in the Department of Thoracic/Head and Neck Medical Oncology. During clinic appointments, potential subjects will be informed of their eligibility and asked if they would be interested in research participation. The attending physician is also available to address any questions or concerns the subject may have. Subjects who agree to participate will sign the protocol-specific informed consent. A research nurse, research data coordinator, or designee with appropriate training and experience sufficient to address issues raised by potential subjects may obtain the Informed Consent.
Ethics oversight	The MD Anderson Cancer Center GEMINI database is prospectively collected from patients consented and enrolled on protocol number PA13-0589 in accordance with the MD Anderson Institutional Review Board. Data collection for Moffitt Cancer Center (MCC) patients was performed under the protocol (MCC 19161), which was formally reviewed and granted approval by MCC in accordance with the Declaration of Helsinki and the 21st Century Cures Act. Both protocols allow for publication of identified data

Note that full information on the approval of the study protocol must also be provided in the manuscript.
